# Cellular retinol binding protein-1 inhibits cancer stemness via upregulating WIF1 to suppress Wnt/β-catenin pathway in hepatocellular carcinoma

**DOI:** 10.1186/s12885-021-08967-2

**Published:** 2021-11-14

**Authors:** Xiangye Liu, Wenhua Shan, Tingting Li, Xiaoge Gao, Fanyun Kong, Hongjuan You, Delong Kong, Shuxi Qiao, Renxian Tang

**Affiliations:** 1 Jiangsu Key Laboratory of Immunity and Metabolism, Department of Pathogenic Biology and Immunology, Xuzhou Medical University, Xuzhou, Jiangsu Province, 221004 People’s Republic of China; 2 Cancer Institute, Xuzhou Medical University, Xuzhou, Jiangsu Province, 221002 People’s Republic of China

**Keywords:** Cellular retinol binding protein-1, Hepatocellular carcinoma, Cancer stemness, Retinoic acid, Wnt inhibitory factor 1, Wnt/β-catenin signaling pathway

## Abstract

**Background:**

CRBP-1, a cytosolic chaperone of vitamin A, is identified in a serious number of cancers; however, its biological role in hepatocellular carcinoma (HCC) needs to be further explored. The aim of our present study is to explore the roles and mechanisms of CRBP-1 in regulating liver cancer by using in vitro and in vivo biology approaches.

**Methods:**

The expression level of CRBP-1 was detected using immunohistochemistry in HCC and matching adjacent non-tumorous liver tissues. Following established stable CRBP-1 overexpressed HCC cell lines, the cell growth and tumorigenicity were investigated both in vitro and in vivo. Intracellular retinoic acid was quantified by ELISA. The relationship between CRBP-1 and WIF1 was validated by using dual luciferase and ChIP analyses.

**Results:**

The low expression of CRBP-1 was observed in HCC tissues compared to the normal liver tissues, while high CRBP-1 expression correlated with clinicopathological characteristics and increased overall survival in HCC patients. Overexpression of CRBP-1 significantly inhibited cell growth and tumorigenicity both in vitro and in vivo. Moreover, overexpression of CRBP-1 suppressed tumorsphere formation and cancer stemness related genes expression in HCC. Mechanically, CRBP-1 inhibited Wnt/β-catenin signaling pathway to suppress cancer cell stemness of HCC. Furthermore, our results revealed that CRBP-1 could increase the intracellular levels of retinoic acid, which induced the activation of RARs/RXRs leading to the transcriptional expression of WIF1, a secreted antagonist of the Wnt/β-catenin signaling pathway, by physically interacting with the region on WIF1 promoter.

**Conclusion:**

Our findings reveal that CRBP-1 is a crucial player in the initiation and progression of HCC, which provide a novel independent prognostic biomarker and therapeutic target for the diagnosis and treatment of HCC.

**Supplementary Information:**

The online version contains supplementary material available at 10.1186/s12885-021-08967-2.

## Background

Cancer stem cells (CSCs) are a small subpopulation of tumor cells, which possess the ability for unlimited self-renewal and differentiation in multiple types of tumors [1]. Growing evidence suggests that CSCs are responsible for tumorigenesis, metastasis, recurrence as well as chemotherapy/radiotherapy resistance, and have been becoming one of the main targets for the current cancer therapy [2]. In recent years, a large number of studies related to CSCs have been performed to identify multiple cell surface proteins as the biomarkers of CSCs [3]. Moreover, it has also been indicated that various developmental and homeostatic stemness signaling pathways are contributed to the self-renewal and differentiation of CSCs [4]. However, the mechanisms of cancer occurrence and development regulated by CSCs are not fully elucidated until now.

Hepatocellular carcinoma (HCC), the most common type of primary liver cancer, is the sixth most commonly diagnosed cancer and the fourth leading cause of cancer-related deaths [5]. Increasing information suggested that liver cancer grew from CSCs which are involved in the initiation and maintenance of malignant tumor, as well as contributing to the resistance against anti-cancer drug [6]. In the year of 2006, suetsugu and collogues firstly isolated high proliferative and tumorigenic capacity CD133^+^ cells as a CSC subpopulation from Huh7 cell lines [7]. After then, a minor subpopulation with cancer stemness properties has been detected in several established HCC cell lines and tumor samples. To date, there have been various accepted cell surface markers used to characterize liver cancer stem cells, including CD13, CD24, CD44, CD90, CD133, EpCAM, OV6, and ALDH [8]. Moreover, signaling pathways as the hallmarks are also important to characterize liver CSCs, including JAK/STAT, Hedgehog, Wnt/β-catenin, Notch, PI3K/PTEN, and NF/κB signaling pathways [8]. Among them, Wnt/β-catenin signaling pathway plays an essential role in self-renewal and maintenance of the stemness properties [9]. Therefore, targeting Wnt/β-catenin signaling pathway will provide numerous numbers of novel targets for exploring an avenue for liver cancer clinical therapeutics.

Retinoic acid (RA), the final biologic active metabolite of vitamin A, is required for lots of essential physiological processes, including morphogenesis, organogenesis, tissue homeostasis, as well as cell growth, differentiation and apoptosis [10]. The biological effects of retinoic acid are achieved through binding to two distinct classes of nuclear receptors: retinoic acid receptors (RARs) and retinoic x receptors (RXRs), which can form heterodimers to regulate the target genes expression via binding to their retinoic acid response elements (RAREs) [11]. Clinical trials have demonstrated that retinoic acid shows potential chemotherapeutic and chemopreventive agent roles in cancer treatment because of its effects to anti-proliferative, apoptotic, and antioxidant [12]. Recently, it has been reported that *all trans*-retinoic acid could inhibit Wnt/β-catenin signaling pathway to suppress the stemness of both embryonic stem cells and CSCs [13, 14]. However, its detailed molecular mechanism has not been well investigated.

Cellular retinol binding protein 1 (CRBP-1), a 15 KDa cytosolic chaperone of vitamin A, is widely expressed in numerous human tissues including liver, kidney, and lung; which is also a master protein to regulate the transportation, metabolism and bioavailability of intracellular vitamin A [15]. Emerging evidence showed that low expression of CRBP-1 was observed in a serious number of human tumors including breast cancer, endometrial cancer, liver cancer, ovarian cancer, and prostate cancer [16–20]. Interestingly, it also highly expressed in astrocytic glioma, lung adenocarcinoma, laryngeal squamous cell carcinoma, tongue squamous cell carcinoma, and oral squamous cell carcinoma [21–25]. Advanced studies showed that high expression of CRBP-1 was relevant to poor prognosis in astrocytic glioma, lung adenocarcinoma, laryngeal squamous cell carcinoma, and tongue squamous cell carcinoma [21–24]. However, the information is limited about the correlation of survival time and expression of CRBP-1 in its low expressed cancers, e.g., breast cancer and liver cancer [16, 18]. Surprisingly, CRBP-1 loss in mammary tissue results in microenvironmental defects similar to those observed at the early stages of tumorigenesis [26, 27], which suggests that CRBP-1 may be a tumor suppressor in its decreased expressed cancers. Unfortunately, there are no more investigations to analyze the role of CRBP-1 in the process of liver cancer occurrence and development.

In the present study, we aim to explore the roles and mechanisms of CRBP-1 in regulating liver cancer by using in vitro and in vivo biology approaches. To the best of our knowledge, this is the first time to evaluate the association between CRBP-1, β-catenin, and CSCs in cancer. We show that overexpression of CRBP-1 induces the intracellular levels of RA to activate RARs/RXRs that transcriptionally promotes the expression of WIF1, a new target gene, then inhibits wnt/β-catenin signaling pathway, thus suppresses the stemness of liver CSCs in HCC.

## Materials and methods

### Patient specimens and immunohistochemistry analysis

A series of 104 HCC tumor specimens were collected from the patients who had undergone surgical resection procedure at the Affiliated Hospital of Xuzhou Medical University. The clinicopathologic information of patients was obtained from the Affiliated Hospital of Xuzhou Medical University Medical Records Department and informed consent was obtained from all patients. Survival time was calculated from the date of surgery to the date of death or to the latest follow-up. All procedures in this study involving human participants were in accordance with the ethical standards of the institutional and/or national research committees and with the 1964 Declaration of Helsinki and its later amendments or comparable ethical standards. The Institutional Review Board of Xuzhou Medical University approved the implementation of this study.

Immunohistochemistry analysis was performed as previously described with 104 patient specimens [28]. In brief, tissue specimens were used to construct tissue microarrays (TMAs), and then were cut into 4-μm thick tissue sections. Following incubated overnight at 4 °C with a 1:200 dilution of CRBP-1 antibody, the sections were further incubated with HRP (horseradish peroxidase)-conjugated secondary antibody for 1 h at room temperature. Finally, the sections were analyzed by using Image-Pro Plus software version 7.0 (Media Cybernetics, Inc., MD, USA) after stained with DAB (diaminobenzidine). In addition, 11 pairs of fresh HCC tissues and matching adjacent non-tumorous liver tissues were used for western-blot analysis.

### Patients publicly available datasets

The gene expression profile with a total number of 374 primary HCC cases and 50 normal liver tissue samples was retrieved from The Cancer Genome Atlas (TCGA) project. And FPKM (Fragments Per Kilobase of transcript per Million mapped reads) was used as a unit representing the gene expression levels. The related clinicopathological information for each sample could be obtained via GDC Data Portal. These collected data was used to perform the comparison of CRBP-1 expression in HCC tissues and normal liver tissues, as well as correlation analysis between CRBP-1 and WIF1, as well as GSEA analysis, which was performed to assess the impact of CRBP-1 expression in HCC.

### Antibodies and reagents

Antibodies against CD90, CD133, EpCAM, SOX2, OCT4, β-catenin, phosphorylated β-catenin, β-actin, and Tubulin were all purchased from Cell Signaling Technology (Danvers, MA, USA), antibody against CRBP-1 was purchased from Santa Cruz biotechnology (Dallas, TX, USA), antibody against WIF1 was purchased from BD Biosciences (San Jose, CA, USA), antibodies of c-Myc, and CyclinD1 were purchased from Proteintech Group (Wuhan, China), and antibodies of RARα used for Chromatin immunoprecipitation assay was purchased from Abcam (Shanghai, China). MG-132 and retinoic acid (RA) were purchased from Sigma-Aldrich (St. Louis, MO, USA). Sorafenib tosylate (Nexavar) was purchased from Bayer (Shanghai, China). Human retinoic acid ELISA kit was purchased from CUSABIO Technology LLC (Wuhan, China).

### Cell culture and CSCs sorting

The human hepatoma cell lines including HepG2, Hep3B, Huh7, and PLC/PRF/5 were all cultured in DMEM medium supplemented with 10% heat inactivated FBS (fetal bovine serum) purchased from Gibco (Life technologies, Carlsbad, CA, USA). Cultures were maintained at 37 °C in a humidified chamber with 5% CO_2_. The CD133^+^ CSCs were sorted using magnetic beads according to the manufactures’ introductions. In brief, the individual cell suspension of PLC/PRF/5 cells was incubated with magnetic beads conjugated with anti-human CD133 antibodies (Miltenyi Biotec, Germany) for 30 min at 4 °C. Following loaded onto LS columns, CD133^+^ cells were separated using a QuadroMACS™ (Miltenyi Biotec, Germany). The acceptable cells were cultured and used in the following experiments.

### Total RNA isolation, RT-PCR and quantitative-PCR (qPCR) assays

TRIzol reagent (Invitrogen, CA, USA) was used to isolate total RNA from cells and tissues according to the manufacturer’s instructions. The cDNA was generated with random primers and amplified with a One Step PrimeScript RT-PCR Kit (Takara, Dalian, China). qPCR was performed using SYBR® Premix *E*x Taq Kit (Takara, Dalian, China) and ran on the Roche LightCycler®480 system following manufacturer’s instructions. GAPDH was used for normalization of qPCR data. The primer sequences were listed in supplementary Table S1. Relative expression values from three independent experiments were calculated following the 2^−ΔΔCt^ method [29].

### Plasmid construction and virus infection

The cDNA fragment encoding the entire CRBP-1 coding sequence was obtained using RT-PCR amplified with specific primers. Primers contained XbaI and EcoRI restriction sites to assist subsequent cloning into pCDH1-CMV-MCS-EF1-Puro lentivirus vector (System Biosciences, CA, USA). As previously described [30], CRBP-1 overexpression lentivirus were generated by co-transfecting 293 T cells with the packing vectors pSPAX2 and pMD2.G. Stable cell lines overexpressing CRBP-1 were generated by infected lentivirus and screened with 2 μg/ml puromycin for two weeks. The primer sequences for gene cloning are available in supplementary Table S1.

### Cell proliferation, cell cytotoxicity and colony formation assays

Cell proliferation was detected with a MTT assay kit (KeyGEN Biotech, Nanjing, China) according to manufacturer’s instructions. For cytotoxicity assay, the cells were treated with indicated concentrations of sorafenib for 48 h, and then cell viability was measured with a MTT assay kit. Half maximal inhibitory concentration (IC_50_) values were calculated from dose-response curves. For colony formation assay, the equal number of CRBP-1 overexpressed and control cells were allowed to sub-culture and grow in 6-well plates for two weeks, and then the number of colonies was counted under a light microscope following stained with 0.1% crystal violet.

### Tumorsphere formation assay

Two thousand cells were plated in 6-well ultra-low attachment plate (Corning, NY, USA) and grown in serum-free DMEM/F12 medium (Thermo Fisher Scientific, MA, USA) supplemented with 20 μg/ml B27 (Invitrogen, CA, USA), 20 ng/ml EGF (BD Biosciences, NJ, USA), and 20 ng/ml bFGF (BD Biosciences, NJ, USA) at 37 °C and 5.0% CO_2_. Fresh media was added every 3 days. After 7–10 days, the number of tumorsphere, which presented as tight, spherical, non-adherent masses and more than 50 μm in diameter, was calculated, and images of the tumorsphere were captured under an inverse microscope.

### Protein preparation and western-blot

Both fresh tissues and cultured cells were homogenized and lysed in RIPA buffer containing protease inhibitors. The supernatant was collected after centrifuging at 13,000 g for 15 min at 4 °C, and total protein concentration was determined with a BCA protein concentration assay kit (Beyotime, Shanghai, China). Equal quantities of obtained protein samples were separated by sodium dodecyl sulfate polyacrylamide gel electrophoresis (SDS-PAGE) and transferred to polyvinylidene difluoride (PVDF) membranes. After blocking with 5% nonfat-dried milk dissolved in TBST buffer (TBS containing 0.1% Tween-20), the membranes were further incubated with primary antibodies overnight at 4 °C. Then membranes were incubated with secondary antibodies at room temperature for 1 h. Immunoreactive bands were visualized using a Tanon imaging system (Tanon Science & Technology Co., Ltd., Shanghai, China).

### RNA-seq and differentially expressed gene analysis

The RNA-sequencing for the CRBP-1 overexpressed PLC/PRF/5 and control cells (triplicates) was performed using HiSeqX Ten system from illumina by Vazyme Biotech Co., Ltd. (Nanjing, China). After evaluated the sequencing data, transcriptome reads aligned to the reference genome were quantified and normalized to FPKM, differences between CRBP-1 overexpressed and control FPKM values were compared with the software Cufflinks v2.2.1. Transcripts with fold change ≥1.5 and a *p*-value < 0.05 were defined as genes that were differentially expressed between the two groups [31]. Differentially expressed gene level was utilized to investigate CRBP-1 regulating cancer stem cell mechanism on transcriptome.

### Intracellular retinoic acid analysis

Intracellular retinoic acid was measured using a human retinoic acid ELISA kit (CUSABIO, Wuhan, China) following the manufacture’s description. Briefly, the equal number of control and CRBP-1 overexpressed PLC/PRF/5 cells were lysed with ultrasonicator on ice. Subsequently, the supernatant of cells was collected under centrifuging at 10,000 g for 10 min at 4 °C. Equal volume of samples or standard was added into each well of 96-well plates, and then HRP-conjugate was added. Following incubated for 60 min at 37 °C and washed with washing buffer, TMB substrate was added into each well. After further incubation for 20 min at 37 °C, the reaction was stopped with stop solution. Finally, the OD value of each well was measured by using a microplate reader at 450 nm. The concentration of retinoic acid was calculated depending on the standard curve and OD value, all the experiments were performed in triplicate.

### Luciferase reporter assay

Luciferase assays were performed with the Dual-Glo® luciferase assay system (Promega, WI, USA) following the description by manufacturer. In brief, WIF1 promoter reporter containing firefly luciferase and control or CRBP-1 plasmids were co-transfected into 293 T cells by using Lipofectamine 2000 (Invitrogen, CA, USA). Following further 48 h incubated post infection, the monolayer cell culture was harvested and resuspended in passive lysis buffer. Finally, the luciferase signal was monitored by using the Dual-Glo® luciferase reporter assay system and a luminometer (Molecular Devices, CA, USA). The WIF1 promoter reporter activity was shown as the relative ratio of firefly luciferase activity to *Renilla* luciferase activity. And the specific activity was presented as the fold change of CRBP-1 overexpressed group versus the control one. All the experiments were performed in triplicate.

TOP/FOP-Flash luciferase reporter system was used to assess the transcriptional activity of β-catenin. Briefly, 293 T cells were co-transfected with control or CRBP-1 plasmids and TOP-Flash or FOP-Flash by using Lipofectamine 2000 (Invitrogen, CA, USA), respectively. After transfection for 48 h, the cells were harvested. And then, both firefly and *Renilla* luciferase activity were measured according to above description. The TOP/FOP-Flash values were normalized to *Renilla* luciferase activity. The β-catenin activity was reported as the fold change of TOP/FOP-Flash activity in CRBP-1 overexpressed cells and control cells. All experiments were performed in triplicate.

### Chromatin immunoprecipitation (ChIP) assay

ChIP assay was performed by using a ChIP assay kit (Beyotime, Shanghai, China) according to the manufacturer’s introductions. In brief, approximately 1 × 10^6^ CRBP-1 overexpressed PLC/PRF/5 cells were treated with 1.0% formaldehyde for 15 min at room temperature to cross-link chromatin-associated proteins to DNA. Then, the reaction was quenched with 125 mM glycine for further 10 min at room temperature. Following harvested by gently scraping, cell pellets were re-suspended in 300 μl SDS lysis buffer (1.0% SDS, 1 mM PMSF, and protease inhibitor) and lysed on ice for 30 min. The lysates were sonicated with high power for 30-s pluses to shear the DNA to 200–500 bp fragments. Finally, the lysates were cleared by centrifugation at 13, 000 rpm for 10 min at 4 °C. After diluted with ChIP dilution buffer, the supernatant was pre-cleared with salmon sperm DNA blocked Protein A/G agarose beads. And then, target protein-DNA complexes were immunoprecipitated by incubating with 5 μl anti-RARα antibody or normal rabbit immunoglobulin G as the negative control at 4 °C for overnight. Following incubated with 30 μl Protein A/G agarose beads for further 2 h, the antibody-protein-DNA-bead complex were centrifuged at 1000 g for 1 min at 4 °C. After washes, the pellets were eluted with fresh elution buffer (1% SDS in 0.1 M NaHCO_3_), and the crosslink was reversed with 0.3 M NaCl for 4 h at 65 °C. Finally, the purified and input DNA was used as template for PCR amplification with specific primers listed in supplementary Table S1.

### Subcutaneous xenograft animal model

Female 6–8-week-old Balb/c nude mice were purchased from the Beijing Vital River Laboratory Animal Technology Co., Ltd. (Beijing, China) and maintained at the Xuzhou Medical University Animal Center (Xuzhou, China). The mice were subcutaneously inoculated in the flank with either control or CRBP-1 overexpressed PLC/PRF/5 cells (1 × 10^6^/mouse). The length and width of the tumors were measured with a caliper every three days, and tumor volumes were calculated as: length×width^2^/2. The mice were killed at day 39, and tumors were surgically isolated and photographed. The study concerning mice was carried out in strict accordance with the recommendations of the Xuzhou Medical University Laboratory Animal Ethics Committee Care and Use of Laboratory Animals guidelines. The protocol was approved by the Xuzhou Medical University Laboratory Animal Ethics Committee (Permit Number: 201547).

### Statistical analysis

Wilcoxon signed-rank test was used to analyze mRNA levels of CRBP-1 in HCC tissue samples and normal liver tissue samples. Single variable analysis was performed using a two-sided *Fisher’s* exact test. Multivariate analysis of overall survival odds ratio (OR) and 95% confidence intervals (CI) were performed with Cox proportional-hazards regression. Overall survival analysis for the patients was performed using the Kaplan-Meier method with a log-rank test. The unpaired student’s *t*-test was used to determine the statistical significance of differences between two groups. Data were presented as mean ± standard deviation (SD), *p* < 0.05 was considered statistically significant, data analysis was performed with SPSS software package 16.0 (IBM SPSS Software, Inc., IL, USA) or GraphPad Prism 8.0 (GraphPad Software, Inc., CA, USA). Gene Set Enrichment Analysis (GSEA) was performed using the Bioconductor package clusterProfiler based on KEGG pathways (minimal set size = 15, maximal set size = 500).

## Results

### High CRBP-1 expression positively correlates with clinicopathological characteristics and increased overall survival in HCC patients

**Fig. 1:**
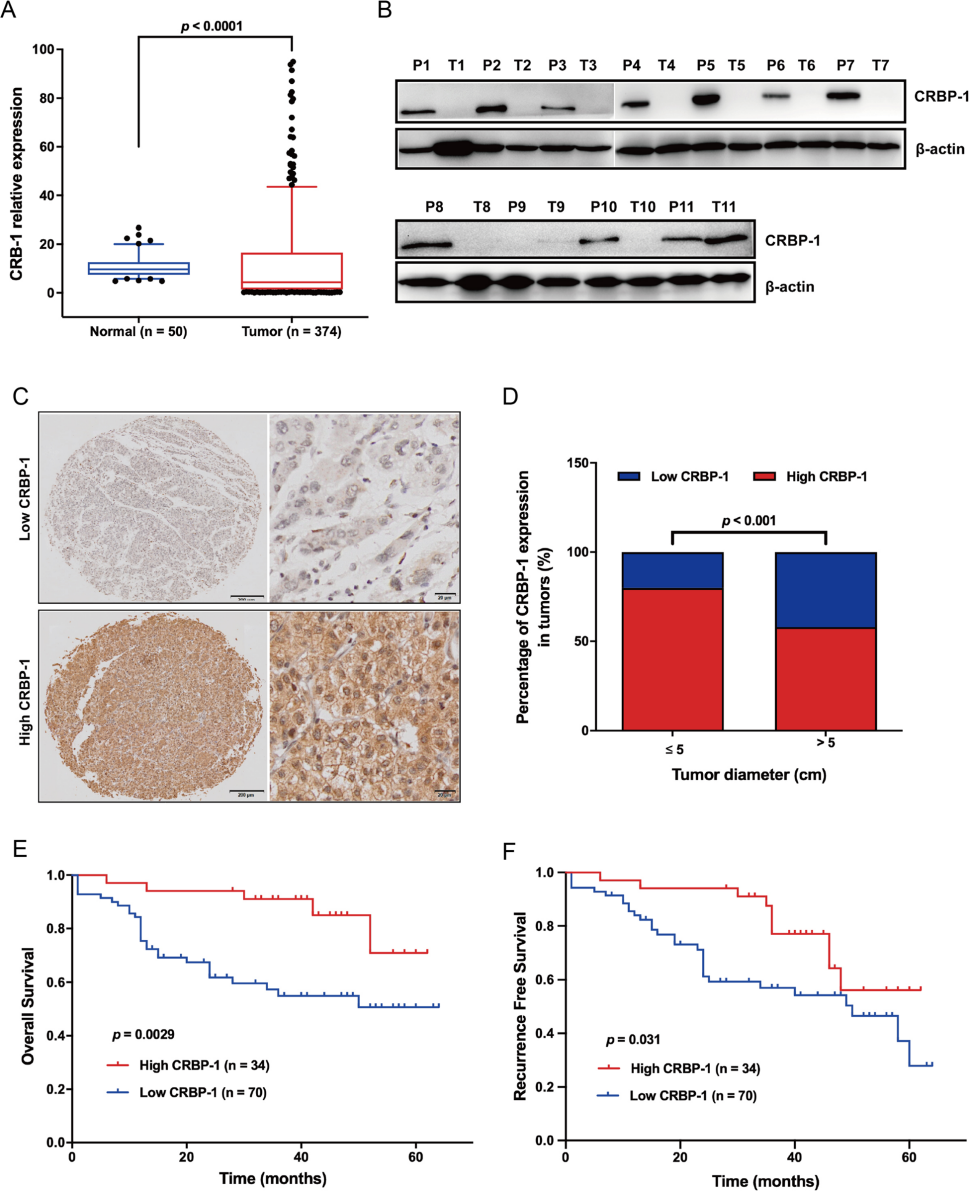
**CRBP-1 high expression is associated with a better outcome for HCC patients.** (A) The mRNA expression of CRBP-1 in normal liver tissue samples (*n* = 50) and HCC tissue samples (*n* = 374), data was obtained from TCGA. Wilcoxon signed-rank test was used to evaluate the significance for CRBP-1 expression in normal and HCC samples, data shown represent box and whisker plots (bar = median, box = interquartile range, whiskers = 10–90 percentile). (B) The protein expression of CRBP-1 in HCC tissue samples (T) and their matched para-carcinoma tissues (P) was detected by using western-blot. (C) Immunohistolochemical analysis of CRBP-1 protein expression in HCC tissue microarray sliders. Representative images of low expression (upper, *n* = 70) and high expression (under, *n* = 34) of CRBP-1 in HCC tissue samples. (D) The analysis of correlation between CRBP-1 expression in HCC tissue samples and tumor size. Data was obtained from 104 HCC patients, and the expression level of CRBP-1 was detected by immunohistolochemical analysis. Two-sided *Fisher*’s exact test was used to evaluate the significance, and *p* < 0.05 was considered to be significant. (E and F) Kaplan-Meier analysis of CRBP-1 expression was performed on the overall survival and recurrence free survival of 104 HCC patients. Log-rank test was used to evaluate the significance, and *p* < 0.05 was considered to be significant

In order to characterize the role of CRBP-1 in HCC, we firstly evaluated the CRBP-1 gene expression in HCC samples (*n* = 374) and normal liver tissue samples (*n* = 50), which were obtained from the TCGA database. Our results showed that the mRNA levels of CRBP-1 were significantly lower in human HCC samples than that in normal liver tissues (Fig. 1A). Furthermore, we identified the expression levels of CRBP-1 in human HCC tissues (T) and para-carcinoma tissues (P) by using western-blot assay with 11-paired fresh HCC tissues and their matched para-carcinoma tissues. As shown in Fig. 1B, CRBP-1 was nearly undetectable in HCC tissues, whereas this protein was remarkably expressed in their matched para-carcinoma tissues. Moreover, we performed an immunohistochemical analysis of 104 paraffin-embedded HCC tissues in TMA (tissue microarray) sliders to investigate the expression levels of CRBP-1. Our results revealed that 67.3% (70/104) of HCC patients showed relatively low CRBP-1 expression, whereas 32.7% (34/104) of HCC patients exhibited high CRBP-1 expression (Fig. 1C and Table 1).

**Table 1:** Correlation between CRBP-1 expression and clinicopathological variables in 104 HCC patients

Variables	CRBP-1		
	High	Low	***p*** value
All patients	34	70	
Age (years)			0.408^b^
≤ 60	21	40	
> 60	13	30	
Gender			0.570^b^
Males	27	58	
Females	7	12	
AFP (ng/mL)			0.369^b^
≤ 400	17	28	
> 400	17	42	
Pathological stage			0.049^b^
High	8	15	
Middle	23	34	
Low	3	21	
Tumor size (cm)			< 0.001^b^
≤ 5	27	29	
> 5	7	41	
Child grade			
A	28	57	0.570^b^
B	6	13	
ALT (U/L)	71.9 ± 10.8	77.9 ± 7.19	0.638^a^
AST (U/L)	76.3 ± 12.9	74.6 ± 6.21	0.891^a^
PLT (10^9^/L)	185.5 ± 14.3	175.8 ± 7.77	0.518^a^
ALB (g/L)	37.1 ± 2.07	37.9 ± 1.27	0.748^a^

^a^ Statistical analysis was performed with Mann-Whitney U test. ^b^ Statistical analysis was performed with *Fisher’s* exact probability test

Furthermore, we also analyzed the correlation between CRBP-1 expression and clinicopathological characteristics in 104 HCC specimens. The results showed that CRBP-1 expression was significantly associated with pathological stage and tumor size (Table 1). Interestingly, high CRBP-1 expression was significantly correlated with small tumor size (*p* < 0.001, Fig. 1D). However, CRBP-1 expression was not significantly associated with clinicopathogical characteristics including age, gender, Child-Grade, AFP, ALT, AST, PLT, or ALB (Table 1). The results of Kaplan-Meier survival analysis revealed that patients with high CRBP-1 expression in HCC had a significantly higher overall survival compared with those with low CRBP-1 expression (*p* = 0.0029, Fig. 1E). Moreover, HCC patients with high CRBP-1 expression had better recurrence free survival than those with low CRBP-1 expression (*p* = 0.031, Fig. 1F).

In addition, to evaluate the independent prognostic value of CRBP-1 expression levels for HCC patients, univariate and multivariate Cox progression analysis was performed. The univariate Cox regression analysis results reveled that CRBP-1 expression as an independent prognostic marker for HCC patient’s overall survival (OR: 0.342, 95% CI: 0.150–0.784, *p* = 0.011; Table 2) and recurrence free survival (OR: 0.464, 95% CI: 0.226–0.955, *p* = 0.037; Table 2). Moreover, our multivariate Cox regression analysis results showed that CRBP-1 expression was also an independent prognostic marker for both overall survival (OR: 0.493, 95% CI: 0.203–1.197, *p* = 0.018; Table 3) and recurrence free survival (OR: 0.611, 95% CI: 0.279–1.341, *p* = 0.019; Table 3). To sum up, our results indicated that CRBP-1 was lowly expressed in HCC tissues and high expression of CRBP-1 in HCC patients positively prognosing good overall survival and recurrence free survival, which suggest that it may suppress the initiation and development of HCC.

**Table 2:** Univariate Cox regression analysis for overall survival (OS) and recurrence free survival (RFS) in 104 HCC patients

Variables	OS		RFS	
	OR (95%CI)	***p*** value	OR (95%CI)	***p*** value
Age (≤ 60 vs. > 60 years)	1.206 (0.631–2.303)	0.571	0.989 (0.524–1.864)	0.972
Gender (males vs. females)	1.033 (0.454–2.352)	0.938	1.074 (0.495–2.334)	0.856
Pathological stage (high and/or middle vs. low)	3.819 (2.116–6.893)	< 0.001	3.172 (1.779–5.658)	< 0.001
Tumor diameter (≤ 5 vs. > 5 cm)	1.919 (1.000–3.681)	0.050	2.157 (1.152–4.039)	0.016
AFP (> 400 vs. < 400 ng/ml)	2.767 (1.305–5.868)	0.008	1.165 (0.621–2.186)	0.634
Child-Pugh grade (A vs. B)	1.848 (0.893–3.821)	0.098	0.899 (0.377–2.144)	0.810
Tumor Recurrence (yes vs. no)	3.894 (1.983–7.646)	< 0.001	–	–
CRBP-1 (high vs. low)	0.342 (0.150–0.784)	0.011	0.464 (0.226–0.955)	0.037

*p* values were calculated with log-rank test

**Table 3:** Multivariate Cox regression analysis for overall survival (OS) and recurrence free survival (RFS) in 104 HCC patients

Variables	OS		RFS	
	OR (95%CI)	***p*** value	OR (95%CI)	***p*** value
Age (≤ 60 vs. > 60 years)	1.528 (0.746–3.130)	0.246	0.848 (0.437–1.645)	0.627
Gender (males vs. females)	1.109 (0.439–2.790)	0.827	1.795 (0.745–4.325)	0.192
Pathological stage (high and/or middle vs. low)	3.411 (1.689–6.888)	< 0.001	2.785 (1.591–4.877)	< 0.001
Tumor diameter (≤ 5 vs. > 5 cm)	0.913 (0.403–2.071)	0.828	1.769 (0.846–3.699)	0.130
AFP (> 400 vs. < 400 ng/ml)	3.346 (1.483–7.550)	0.004	0.911 (0.470–1.764)	0.782
Child-Pugh grade (A vs. B)	2.993 (1.366–6.556)	0.006	1.042 (0.431–2.521)	0.927
Tumor Recurrence (yes vs. no)	4.507 (2.082–9.756)	< 0.001	–	–
CRBP-1 (high vs. low)	0.493 (0.203–1.197)	0.018	0.611 (0.279–1.341)	0.019

*p* values were calculated with log-rank test

### CRBP-1 inhibits cell growth in vitro and tumorigenicity in vivo of HCC

**Fig. 2:**
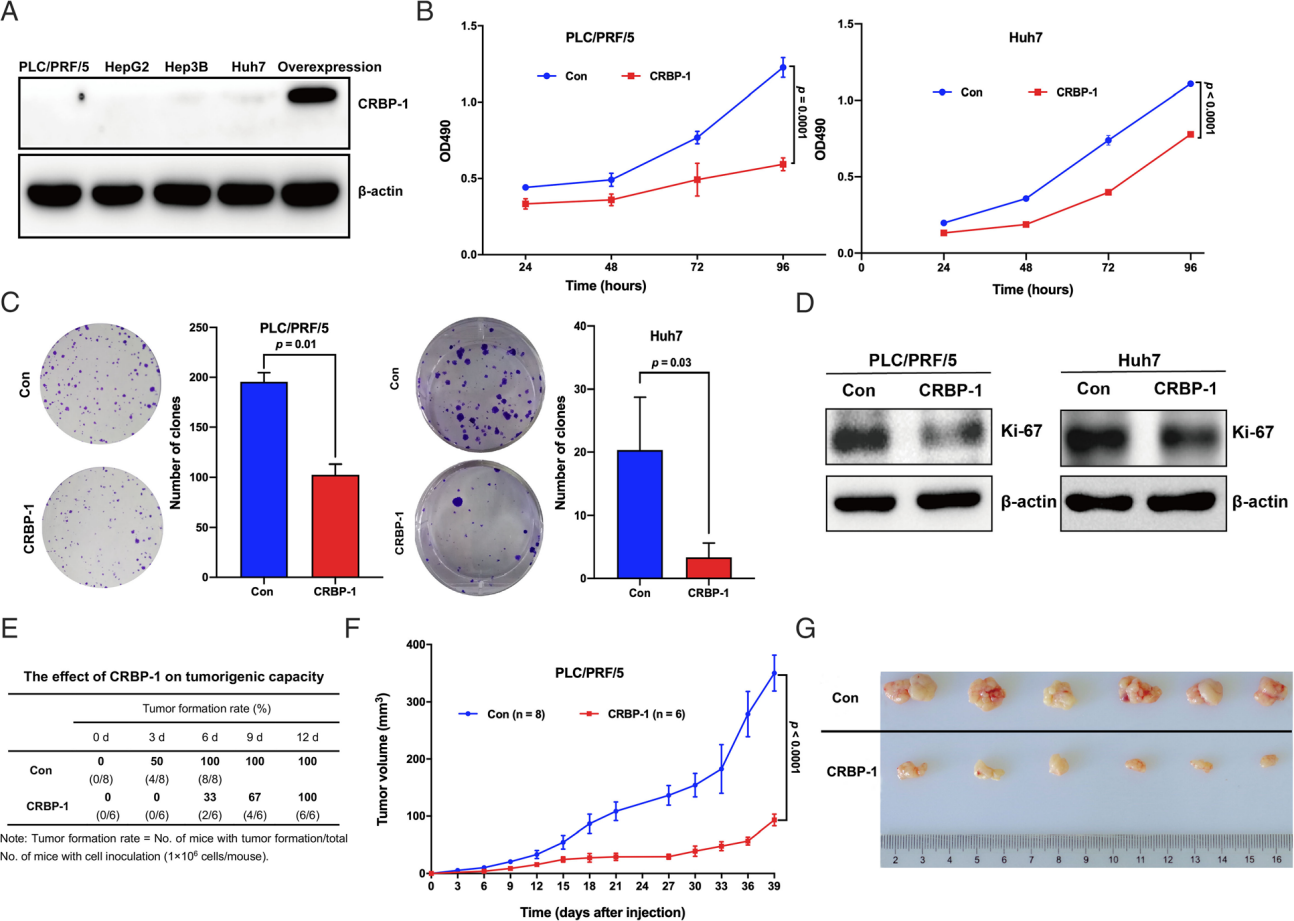
**CRBP-1 contributes to the cell growth in vitro and tumorigenicity in vivo of HCC.** (A) Western-blot analysis of the expression of endogenous CRBP-1 in HCC cell lines. (B) Cell proliferation analysis of CRBP-1 overexpressed and control PLC/PRF/5 and Huh7 cells. All data shown represent the mean ± SD from three independent experiments. (C) Colony formation analysis of CRBP-1 overexpressed and control PLC/PRF/5 and Huh7 cells. All data shown represent the mean ± SD from three independent experiments. (D) Western-blot analysis of the expression of Ki-67 in CRBP-1 overexpressed and control PLC/PRF/5 and Huh7 cells. (E) The tumor formation rate of CRBP-1 overexpressed and control PLC/PRF/5 cells inoculated in Balb/c nude mice. (F) The tumor growth curve was shown in CRBP-1 overexpressed PLC/PRF/5 cells and control ones. All data shown represent the mean ± SD from indicated numbers of xenograft tumor. (G) The photograph of xenograft tumors respectively derived from CRBP-1 overexpressed and control PLC/PRF/5 cells inoculated in Balb/c nude mice

To investigate CRBP-1 expression in liver cancer cells, the expression of CRBP-1 in HepG2, Hep3B, Huh7, and PLC/PRF/5 cell lines was detected by using western-blot. Our results showed that low expression of CRBP-1 was observed in all four cell lines (Fig. 2A). Furthermore, PLC/PRF/5 and Huh7 cells were created to stably overexpressed CRBP-1 using lentiviral overexpression system. Then cell proliferation and colony formation analyses were performed to investigate the cell growth and viability after CRBP-1 overexpression. Our results showed that cell growth and cell viability were significantly inhibited in CRBP-1 overexpressed PLC/PRF/5 and Huh7 cells compared to the control groups (Fig. 2B and C). In addition, overexpression of CRBP-1 obviously decreased the expression of Ki-67 in both PLC/PRF/5 and Huh7 cells, which is a classical proliferation marker for human tumor cells (Fig. 2D). Subsequently, 1 × 10^6^ PLC/PRF/5 cells were inoculated into Balb/c nude mouse, and then the rate of tumor formation as well as tumor growth were monitored every three days until the end of the experiment. Our results presented that the rate of tumor formation was decreased at CRBP-1 overexpressed xenografts compared to the controls (Fig. 2E). In addition, the tumor volume was also obviously smaller in CRBP-1 overexpressed xenografts compared to the control groups (Fig. 2F and G). Altogether, our results suggested that CRBP-1 inhibited the cell growth and proliferation in vitro and tumor formation of HCC in vivo*.*

### CRBP-1 suppresses cancer stemness properties of HCC

**Fig. 3:**
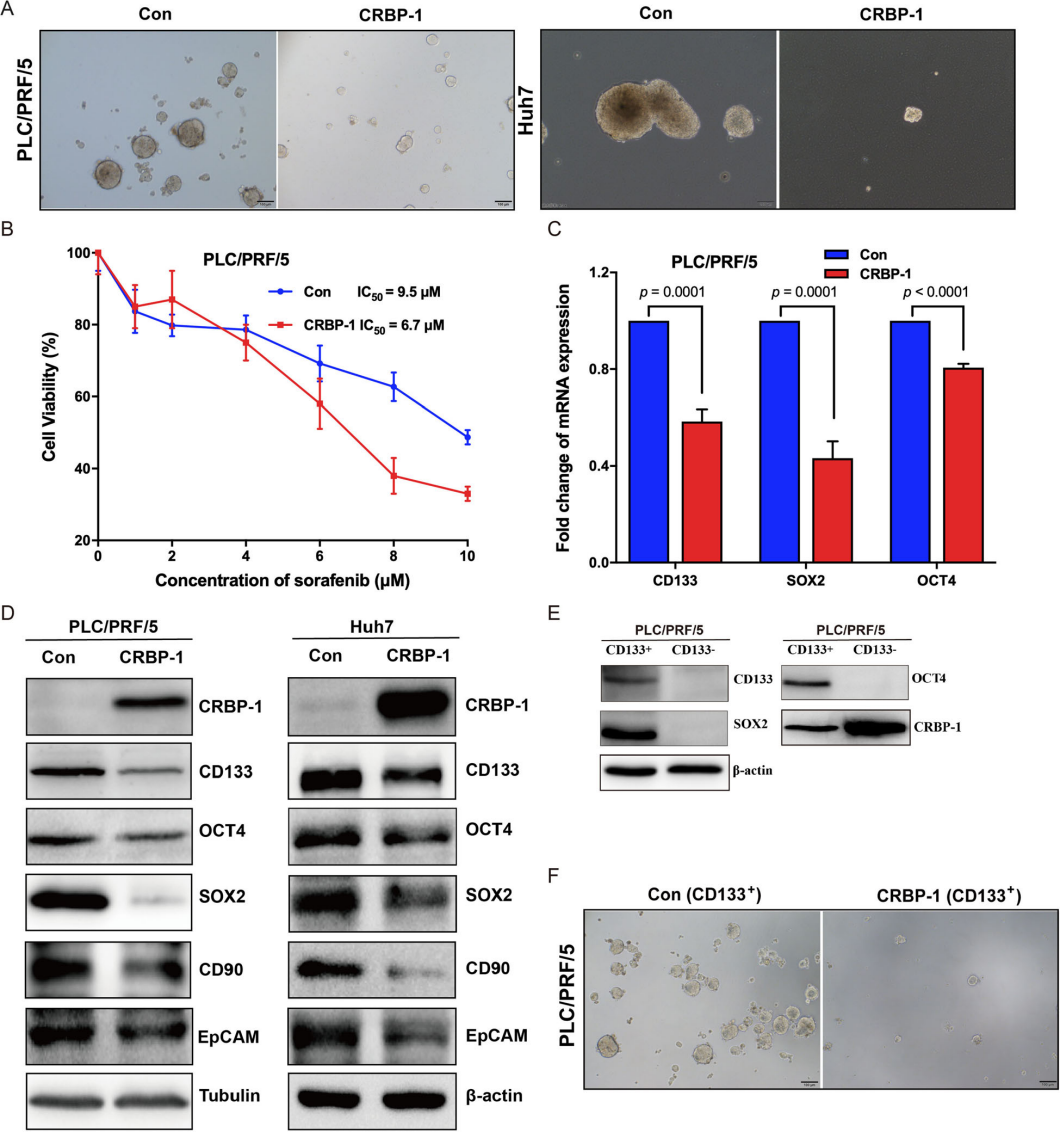
**CRBP-1 regulates cancer stemness properties of HCC.** (A) Representative images of tumorspheres formed by CRBP-1 overexpressed PLC/PFR/5 and Huh7 cells as well as control ones. (B) Cell viability of CRBP-1 overexpressed PLC/PFR/5 cells and control ones after treated with different concentrations of sorafenib for 48 h. All data shown represent the mean ± SD from three independent experiments. IC_50_ values were calculated from dose-response curves. (C) qPCR analysis of the expression of cancer stem cell markers including CD133, SOX2, and OCT4 in CRBP-1 overexpressed PLC/PRF/5 cells and control cells. Data shown for represent the mean ± SD from three independent experiments. (D) Western-blot analysis of the expression of cancer stem cell markers including CD133, OCT4, SOX2, CD90, and EpCAM in CRBP-1 overexpressed PLC/PRF/5 and Huh7 cells as well as control cells. (E) Western-blot analysis of the expression of cancer stem cell markers including CD133, SOX2, and OCT4 in CRBP-1 overexpressed PLC/PRF/5 CD133^+^ cells and CD133^−^ cells. (F) Representative images of tumorspheres formed by CRBP-1 overexpressed PLC/PFR/5 CD133^+^ cells and control CD133^+^ cells

Generally, tumorigenesis is driven by a small subset of cancer stem cells (CSCs), self-renewal is critical character of CSCs [32]. In order to characterize the effects of CRBP-1 to the self-renewal ability of CSCs, CRBP-1 overexpressed PLC/PRF/5 and Huh7 cells were cultured in cancer stem cell culture medium for growing tumor sphere. Our results showed that the number and size of tumor spheres of both PLC/PRF/5 and Huh7 cells were inhibited by overexpression of CRBP-1 (Fig. 3A), which suggested that CRBP-1 suppressed the self-renewal ability of CSCs. In addition, CSCs are considered as the main factor for chemotherapy resistance and following cancer recurrence [1]. In order to identify the role of CRBP-1 in cancer cell chemotherapy resistance, the first-line treatment drug for advanced HCC—sorafenib was used to treat CRBP-1 overexpressed PLC/PRF/5 cells. Our results showed that CRBP-1 overexpression decreased the IC_50_ value of sorafenib compared to the controls (Fig. 3B). Altogether, all these results suggested that CRBP-1 suppressed the cell growth and tumorigenicity via inhibiting cancer cell stemness.

To confirm the role of CRBP-1 in the process of regulating HCC cancer cell stemness, mRNA levels of stem cell markers CD133, OCT4 and SOX2 was found dramatically decreased in CRBP-1 overexpressed PLC/PRF/5 cells compared to the control by using qPCR (Fig. 3C). To validate the findings based on qPCR results, our western-blot results also confirmed the remarkably downregulation at the protein levels of these three markers as well as CD90 and EpCAM, another two CSCs maker of HCC, based on CRBP-1 overexpression in both PLC/PRF/5 and Huh7 cells (Fig. 3D). Furthermore, we sorted CD133^+^ and CD133^−^ cells from CRBP-1 overexpressed PLC/PRF/5 cells with magnetic beads. Our results showed that CD133^+^ cells highly expressed OCT4 and SOX2 as usual, but possessed remarkably low expression of CRBP-1 compared to CD133^−^ cells (Fig. 3E). To further investigate the effect of CRBP-1 on cancer stemness, tumorspheres formation assay was performed using CD133^+^ cells separated from PLC/PRF/5 cells with or without CRBP-1 overexpression, respectively. Our results showed that CD133^+^ cells with CRBP-1 overexpression exhibited lower pluripotency compared to the cells without CRBP-1 expression (Fig. 3F). In summary, all our data indicated that CRBP-1 displayed suppression ability to the CSC characteristics in HCC.

### CRBP-1 inhibits Wnt/β-catenin pathway via upregulating WIF1 to suppress cancer cell stemness

**Fig. 4:**
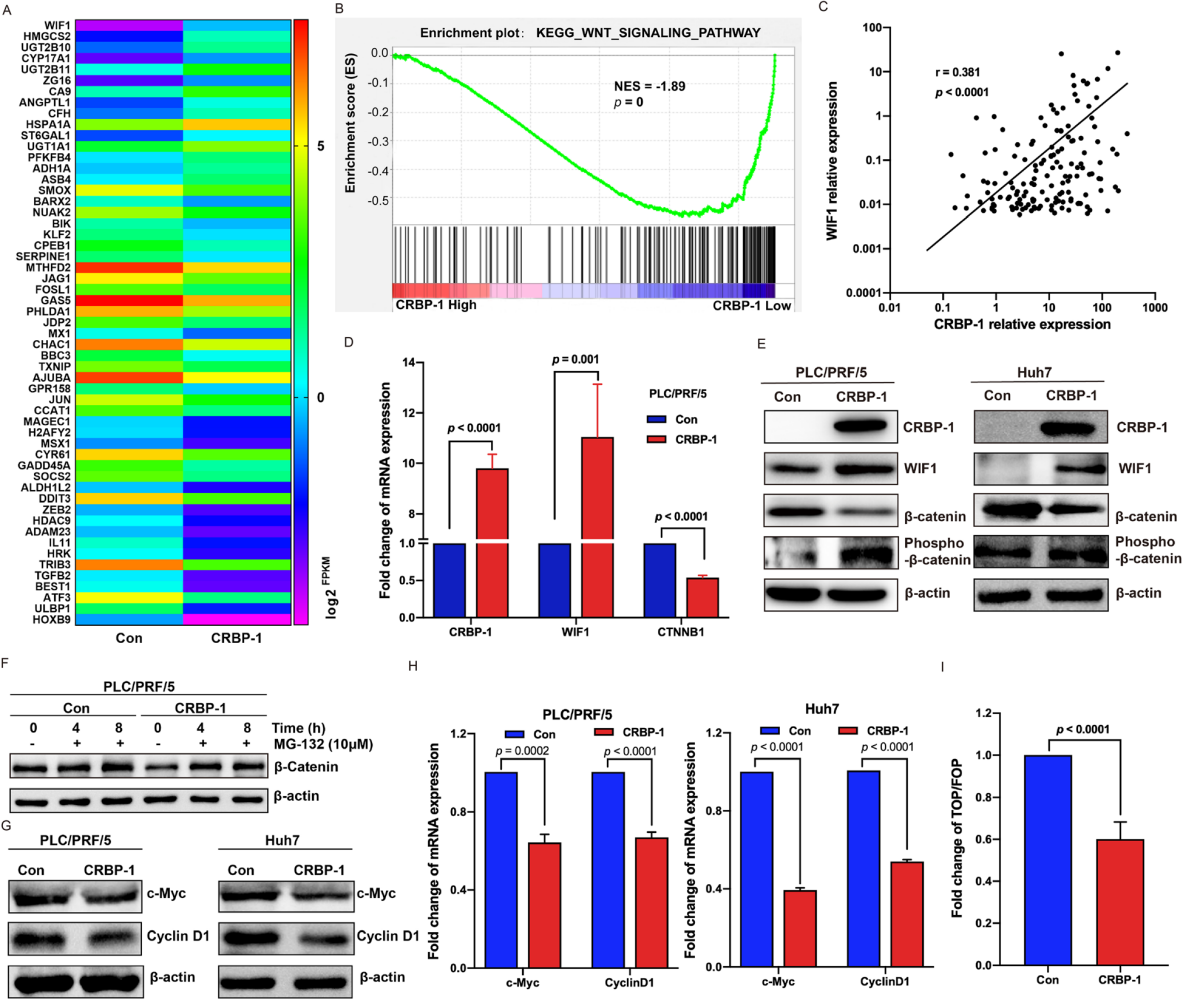
**CRBP-1 inhibits Wnt/β-catenin signaling pathway by upregulating WIF1.** (A) Heatmap of RNA-seq analysis for differentially expressed cancer stemness related genes (fold change ≥1.5, *p* < 0.05) in response to CRBP-1 overexpression. (B) Gene set enrichment analysis (GSEA) was performed to investigate the activation of Wnt/β-catenin signaling pathway against CRBP-1 expression in HCC tissue samples. Dataset were obtained from TCGA (*n* = 374). (C) Correlation analysis between CRBP-1 and WIF1 in HCC tissue samples was performed with Spearman correlation analysis. Dataset were obtained from TCGA (n = 374). (D) qPCR analysis of the expression of WIF1 and β-catenin (CTNNB1) in CRBP-1 overexpressed PLC/PRF/5 cells and control cells. Data shown represent the mean ± SD from three independent experiments. (E) Western-blot analysis of the expression of WIF1, β-catenin, and phosphorylated β-catenin in CRBP-1 overexpressed PLC/PRF/5 and Huh7 cells as well as control cells. (F) Western-blot analysis of the expression of β-catenin in CRBP-1 overexpressed PLC/PRF/5 cells and control cells, which were treated with 10 μM of MG-132 under indicated time. (G) Western-blot analysis of the expression of c-Myc and Cyclin D1 in CRBP-1 overexpressed PLC/PRF/5 and Huh7 cells as well as control cells. (H) qPCR analysis of the expression of c-Myc and Cyclin D1 in CRBP-1 overexpressed PLC/PRF/5 and Huh7 cells as well as control cells. Data shown represent the mean ± SD from three independent experiments. (I) The transcriptional activity analysis of β-catenin in CRBP-1 overexpressed cells and control cells by using TOP/FOP-Flash luciferase reporter system. Data shown represent the mean ± SD from three independent experiments

To explore the mechanism of CRBP-1 suppressing cancer stemness properties of HCC, we performed RNA-seq with CRBP-1 overexpressed and control PLC/PRF/5 cells. After evaluated and analyzed the sequencing data as described in material and methods, differentially expressed genes were characterized including 78 upregulated and 158 down regulated genes (Supplementary Table S2). Interestingly, 15 CSCs-related genes were upregulated in response to CRBP-1 overexpression. Among them, WIF1 (Wnt inhibitory factor 1) was the highest upregulated one (Fig. 4A), which can bind to Wnt proteins and prevent them from triggering Wnt/β-catenin signaling pathway that plays an essential role in maintenance of the stemness properties [33]. Accordingly, we hypothesized that CRBP-1 may have an impact on Wnt/β-catenin signaling pathway to suppress cancer cell stemness in HCC.

To assess our hypothesis, Gene set enrichment analysis (GSEA) was performed to identify the pathways that were affected by CRBP-1 expression in human HCC samples, which were obtained from TCGA database. Finally, 46 pathways were found to be suppressed correlated CRBP-1 expression (*p* < 0.05, Supplementary Table S3). Interestingly, the Wnt signaling pathway showed strong suppression upon CRBP-1 high expression (Fig. 4B). Moreover, Spearman correlation analysis was performed to evaluate the gene expression interaction of CRBP-1 and WIF1 in 374 pairs of HCC samples from TCGA. As shown in Fig. 4C, strong co-expression between CRBP-1 and WIF1 in HCC was observed with a correlation coefficient r = 0.381 (*p* < 0.0001). Importantly, as an endogenous antagonist of Wnt/β-catenin signaling pathway, WIF1 can induce β-catenin degradation [34].

Furthermore, our qPCR results showed that the expression of WIF1 was remarkably promoted, and the expression of β-catenin (CTNNB1) was obviously inhibited in CRBP-1 overexpressed PLC/PRF/5 cells compared to the control (Fig. 4D). Western-blot assay also showed that CRBP-1 overexpression upregulated WIF1 but downregulated β-catenin, and the phosphorylation level of β-catenin was correspondingly upregulated in both PLC/PRF/5 and Huh7 cells (Fig. 4E). It has been reported that intracellular phosphorylated β-catenin was usually degraded via ubiquitin-dependent proteasomal pathway, which could be effectively blocked by a proteasome inhibitor—MG-132 [35]. Then, CRBP-1 overexpressed and control PLC/PRF/5 cells were respectively treated with MG-132 under indicated time. As shown in Fig. 4F, the intracellular level of β-catenin was consistently reduced by CRBP-1 overexpression, which was reversed by additional treatment of MG-132. Additionally, overexpression of CRBP-1 repressed the expression of β-catenin-dependent genes, including c-Myc, CyclinD1 in both PLC/PRF/5 and Huh7 cells at protein and mRNA level (Fig. 4G and H). To further assess the transcriptional activity of β-catenin, TOP/FOP-Flash luciferase reporter system was induced. As shown in Fig. 4I, the fold change of TOP/FOP-Flash was significantly lower in CRBP-1 overexpressed cells than that in control cells. In summary, these results indicated that CRBP-1 inhibited Wnt/β-catenin pathway via upregulating WIF1, then suppressed cancer stemness properties in HCC.

### CRBP-1 activates RARs/RXRs via upregulating intracellular RA level

**Fig. 5:**
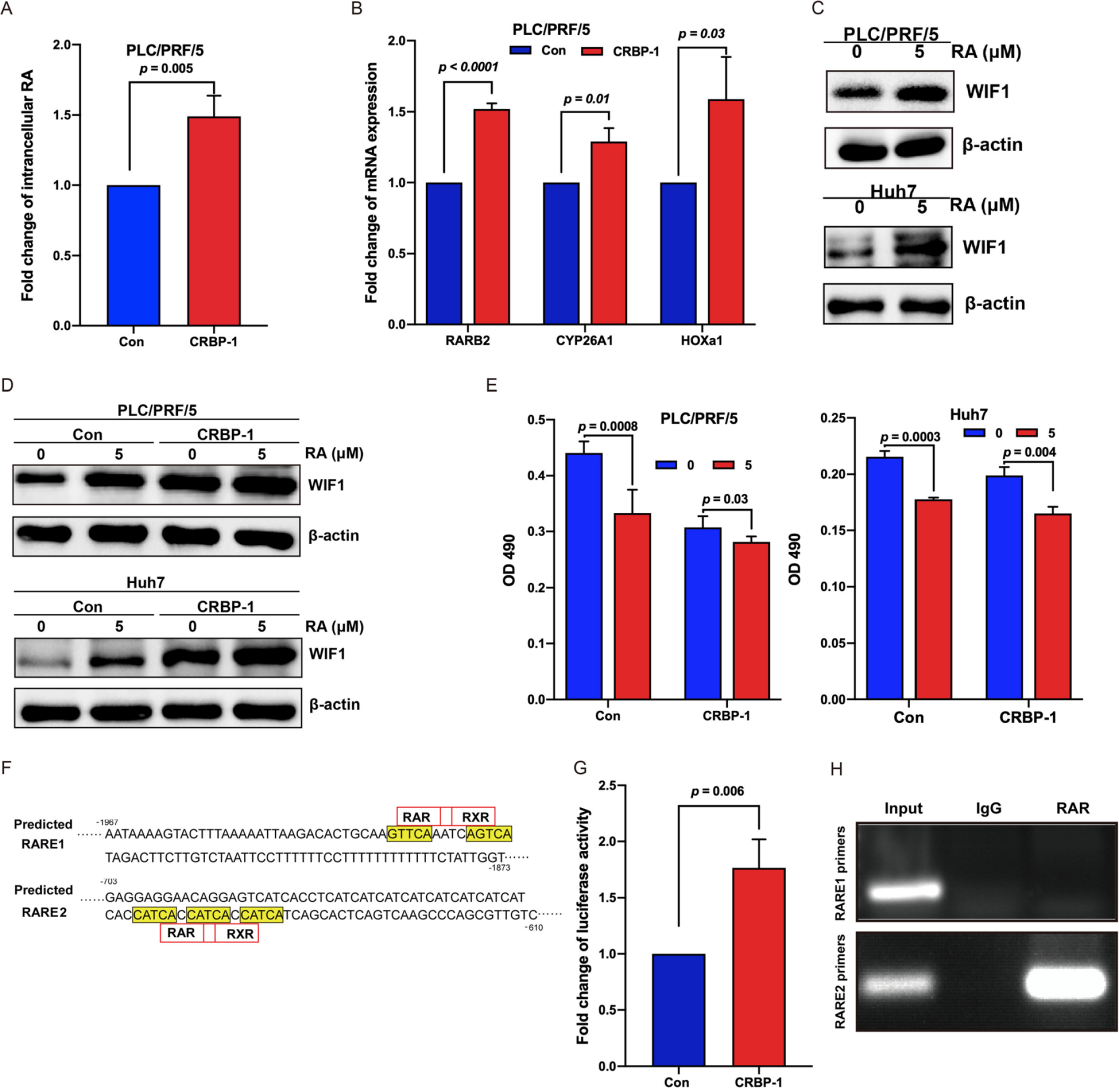
**CRBP-1 regulates WIF1 expression through indirectly activating its promoter transcription.** (A) The intracellular RA analysis in CRBP-1 overexpressed PLC/PRF/5 cells and control cells. All data shown represent the mean ± SD from three independent experiments. (B) qPCR analysis of the expression of representative RAR/RXR target genes including RARB2, CYP26A1, and HOXa1 in CRBP-1 overexpressed PLC/PRF/5 cells and control cells. Data shown represent the mean ± SD from three independent experiments. (C) Western-blot analysis of the expression of WIF1 in PLC/PRF/5 and Huh7 cells after treated with/without 5 μM RA for 48 h. (D) Western-blot analysis of the expression of WIF1 in CRBP-1 overexpressed PLC/PRF/5 and Huh7 cells as well as control cells after treated with/without 5 μM RA for 48 h. (E) Cell proliferation analysis of CRBP-1 overexpressed PLC/PRF/5 and Huh7 cells as well as control cells after treated with/without 5 μM RA for 48 h. Data shown represent the mean ± SD from three independent experiments. (F) The prediction analysis of RAREs in the promoter region of WIF1. (G) The dual-luciferase activity analysis for WIF1 promoter in 293 T cells in response to CRBP-1 overexpression. Data shown represent the mean ± SD from three independent experiments. (H) ChIP analysis of RAREs for WIF1 promoter in CRBP-1 overexpressed PLC/PRF/5 cells

CRBP-1, a chaperone of vitamin A, is a crucial player in the process of vitamin A metabolized to retinoic acid (RA) [15]. In order to investigate the effects of CRBP-1 on vitamin A metabolism, the intracellular level of RA was measured by using ELISA. Interestingly, our results showed that the intracellular level of RA was significantly increased in CRBP-1 overexpressed PLC/PRF/5 cells compared to the controls (Fig. 5A). In addition, it is well known that RA could regulate the transcription of targeted genes including RARβ (RARB2), Cytochrome P450 26A1 (CYP26A1), and Homeobox A1 (HOXa1) through activating RARs/RXRs [36, 37]. In the present study, our qPCR results also showed that upregulation of all these genes was observed in CRBP-1 overexpressed PLC/PRF/5 cells compared to the control (Fig. 5B). All of which suggested that RARs/RXRs was activated in CRBP-1 overexpressed PLC/PRF/5 cells via upregulating intracellular RA.

### CRBP-1 transcriptionally increases the expression of WIF1 through RARs/RXRs

Furthermore, PLC/PRF/5 and Huh7 cells were treated with/without RA for 48 h, then the expression of WIF1 was analyzed by using western-blot. Our results showed that RA could remarkably induce the expression of WIF1 in both PLC/PRF/5 and Huh7 cells (Fig. 5C). Importantly, RA treatment further promoted the expression of WIF1 and inhibited cell proliferation in CRBP-1 overexpressed PLC/PRF/5 and Huh7 cells compared to the control cells (Fig. 5D and E). It has been demonstrated that RA could activate RARs/RXRs to regulate the target genes expression via binding to RARE in their promoter region [11], which promoted us to examine whether a putative RARE exists in the promoter region of WIF1. Our screening results showed that two putative RAREs (RARE1: 5′-GTTCAAATCAGTCA-3′ and RARE2: 5′-CATCACCATCACCATCA-3′) were characterized in WIF1 promoter region (Fig. 5F), suggesting that WIF1 could be a RARE-responsive gene. These results revealed that CRBP-1 might actively enhance the expression of WIF1 via regulating its promoter region, which was mediated by RA.

In order to confirm this hypothesis, we performed dual-luciferase assay to explore whether CRBP-1 enhances the promoter activity of WIF1. The WIF1 promoter-luciferase reporter containing the sequence from − 2000 to + 200 of WIF1 gene promoter region (the start codon ATG is defined as + 1) was constructed. Our results showed that the luciferase activity in CRBP-1 overexpressed cells was significantly higher than that in control cells (Fig. 5G), which suggested that CRBP-1 overexpression could activate the promoter of WIF1. Furthermore, we performed ChIP assay to characterize whether RARα binds to the specific promoter region of WIF1. The specific primers were designed to amplify two putative RAREs in the promoter region of WIF1. Our immunoprecipitation by RARα antibody indicated that enrichment of RARα binds to the RARE2 not RARE1 sequence in the promoter region of WIF1 (Fig. 5H). These data supported our prediction that CRBP-1 transcriptionally increases the expression of WIF1 via activating RARs/RXRs that directly binds to its promoter in HCC.

## Discussion

Cancer stem cells (CSCs) are considered to be a crucial player in liver cancer initiation, metastasis, recurrence, and chemoresistance [38]. Interestingly, the function of CSCs is regulated by altering the activities of several signaling pathways, such as the Hedgehog, Wnt/β-catenin, Notch, and NF/κB pathways, which ultimately affect the occurrence and development of liver cancer [8]. In recent years, some approaches, which attempt to inhibit key CSCs signaling pathways, ablate CSCs using their surface marker antibodies and target quiescent CSCs, have been developed to eliminate this cancer cell subpopulations [1]. Theoretically, targeting CSCs could eradicate tumors; however, there are complex crosstalk amongst gene regulation pathways in tumors, which blockade the implementation of these approaches in clinical treatment [38]. Therefore, there is critical need for exploring novel and effective therapeutic approaches for liver cancer treatment. In the present study, we revealed that CRBP-1 inhibited the stemness properties of HCC via upregulating WIF1, then suppressed Wnt/β-catenin signaling pathway, which provided a novel therapeutic target for clinical management of liver cancer.

To date, retinoic acid (RA) has become an effective treatment for tumor differentiation therapy in the patients acquired acute promyelocytic leukemia (APML), and several solid tumors [36, 39]. However, the usage of RA also presents some drawbacks, such as retinoic acid syndrome (RAS) and drug resistance, which partially caused by aberrant metabolism and uptake of RA [40–42]. Dietary vitamin A is the main source of intracellular RA after two oxidization steps, which is controlled by several enzymes and proteins involved in the transport and catabolism [36]. Therefore, we hypothesized that improving the intracellular levels of RA via targeting the metabolic process of vitamin A could contribute to the treatment of malignancy cancers and prevent side-effect caused by excess RA. From numerous functional proteins related to vitamin A metabolism, CRBP-1 attracted attention, which is a chaperone and charges for the intracellular transportation of vitamin A. Thus far, there has been some investigations about the role of CRBP-1 in cancers [16–25]. However, the expression profile of CRBP-1 in the above-related cancers has shown difference in different types of cancers, which indicates that the function of CRBP-1 in tumorigenesis and cancer development remains discrepancy.

In order to characterize the role of CRBP-1 in HCC initiation and development, we primarily investigated the expression profile of CRBP-1 at transcription and translation levels in HCC. Our data showed that both mRNA and protein levels of CRBP-1 decreased in HCC tissues compared to those in their matched non-tumorous tissues (Fig. 1). Furthermore, high CRBP-1 expression not only positively correlated with overall survival and recurrence free survival of HCC patients, but also presented as an independent prognostic marker (Tables 1, 2, and 3). Although the expression of CRBP-1 has been investigated in HCC [18], it is the first time to characterize its role as a biomarker for the disease prognosis. Subsequently, our in vitro and in vivo data also confirmed that overexpression of CRBP-1 could inhibit the cell growth and tumorigenicity of HCC (Fig. 2). Taken together, our results revealed CRBP-1 as a key suppressor in HCC progression and an independent prognostic predictor for HCC patients.

To elucidate the underlying mechanisms, we then identified the cancer stemness properties and stemness-related gene signature. Of significance, overexpression CRBP-1 inhibited tumorspheres formation ability, increased the sensitivity of cancer cells to antineoplastic drugs, and downregulated the expression of stemness-related genes (Fig. 3). CD133 is a reliable membrane marker for CSCs of HCC, which is widely used to isolate CSCs from different types of cancer cells [43]. Furthermore, our results also showed that the tumorspheres formation ability of CRBP-1 overexpressed PLC/PRF/5 CD133^+^ cells were decreased compared to the control PLC/PRF/5 CD133^+^ cells (Fig. 3). Such findings suggest that CRBP-1 inhibits tumorigenesis via suppressing cancer stemness properties in HCC.

Accumulating evidence indicated that cancer stemness properties of HCC were modulated by several molecules and signaling pathways [8, 44]. To characterize the molecular features of CRBP-1 in inhibiting HCC stemness, RNA-seq was performed and found that CRBP-1 overexpression was associated with the highest upregulation of WIF1 among 15 CSCs-related genes. Furthermore, GSEA results showed that CRBP-1 was also highly correlated with Wnt/β-catenin signaling pathway (Fig. 4). The Wnt/β-catenin signaling pathway, as “canonical” Wnt pathway, is a crucial player in many aspects of physiological and pathophysiological processes [45]. Wnt/β-catenin signaling pathway participates in almost every facet of liver biology, including development, homeostasis, as well as regeneration; moreover, it also plays a critical role in CSCs for maintenance of stemness properties and self-renewal [46, 47]. WIF1, an endogenous secreted antagonist of Wnt/β-catenin signaling pathway, can directly bind to Wnt molecules preventing them from binding to their receptors and then inducing β-catenin degradation [34]. This prevention also can inactivate the downstream target genes of Wnt/β-catenin signaling pathway, including c-Myc and Cyclin D1 [48, 49]. Interestingly, our present data showed that the gene expression of CRBP-1 and WIF1 was positively correlated with each other in HCC tissues, CRBP-1 overexpression could upregulate WIF1 in HCC cell lines. Furthermore, the expression of β-catenin and its downstream target genes including c-Myc and Cyclin D1 was also inhibited by CRBP-1 overexpression. In addition, TOP/FOP-Flash reporter assay showed that CRBP-1 overexpression led to attenuate Wnt/β-catenin signaling activity (Fig. 4). To summarize all the points, our study suggested that CRBP-1 inhibited HCC cancer stemness properties via upregulation of WIF1 then suppressed Wnt/β-catenin signaling pathway. On the contrary, it was reported that overexpression of CRBP-1 could upregulate β-catenin to promote osteogenenesis of mesenchymal stem cells [50]. The different effect of CRBP-1 may be associated with cell type and experimental conditions.

WIF1, as a tumor suppressor, has been observed to be downregulated in several human cancers, such as lung cancer, neuroblastoma, and cervical cancer [33, 51, 52]. Accumulating evidence demonstrated that downregulation of WIF1 was mediated through its promoter methylation [33, 51–55], which reminded that the activity of WIF1 promoter played a critical role in its transcriptional modulation. Our present data showed that overexpression of CRBP-1 could promote intracellular RA levels (Fig. 5). Interestingly, it has also been investigated that RA could regulate the target genes expression via binding to their RARE through activated RARs/RXRs [11]. Surprisingly, we also found that the expression of representative RA target genes including RARB2, CYP26A1, and HOXa1 was upregulated during CRBP-1 overexpression, which suggested that RARs/RXRs was activated in CRBP-1 overexpressed PLC/PRF/5 cells. Furthermore, drug dose of RA also increased the expression of WIF1 and further amplified the action in CRBP-1 overexpressed PLC/PRF/5 and Huh7 cells (Fig. 5). Consequently, our findings suggested that CRBP-1 indirectly promoted the transcription of WIF1 through activated RARs/RXRs binding to its RARE in the promoter regions. Interestingly, CRBP-1 was reported to inhibit RXRα-induced β-catenin degradation in HEK293T cells [50], but the exact mechanism remains unknown. Afterwards, our further studies confirmed that a RARE domain was identified in the promoter region of WIF1 by using dual-luciferase assay and ChIP assay (Fig. 5). To the best of our knowledge, this is the first time to demonstrate that WIF1 is the target gene of RARs/RXRs. These results explained the reason that the expression of WIF1 could be regulated by CRBP-1 to some extent.

## Conclusions

**Fig. 6:**
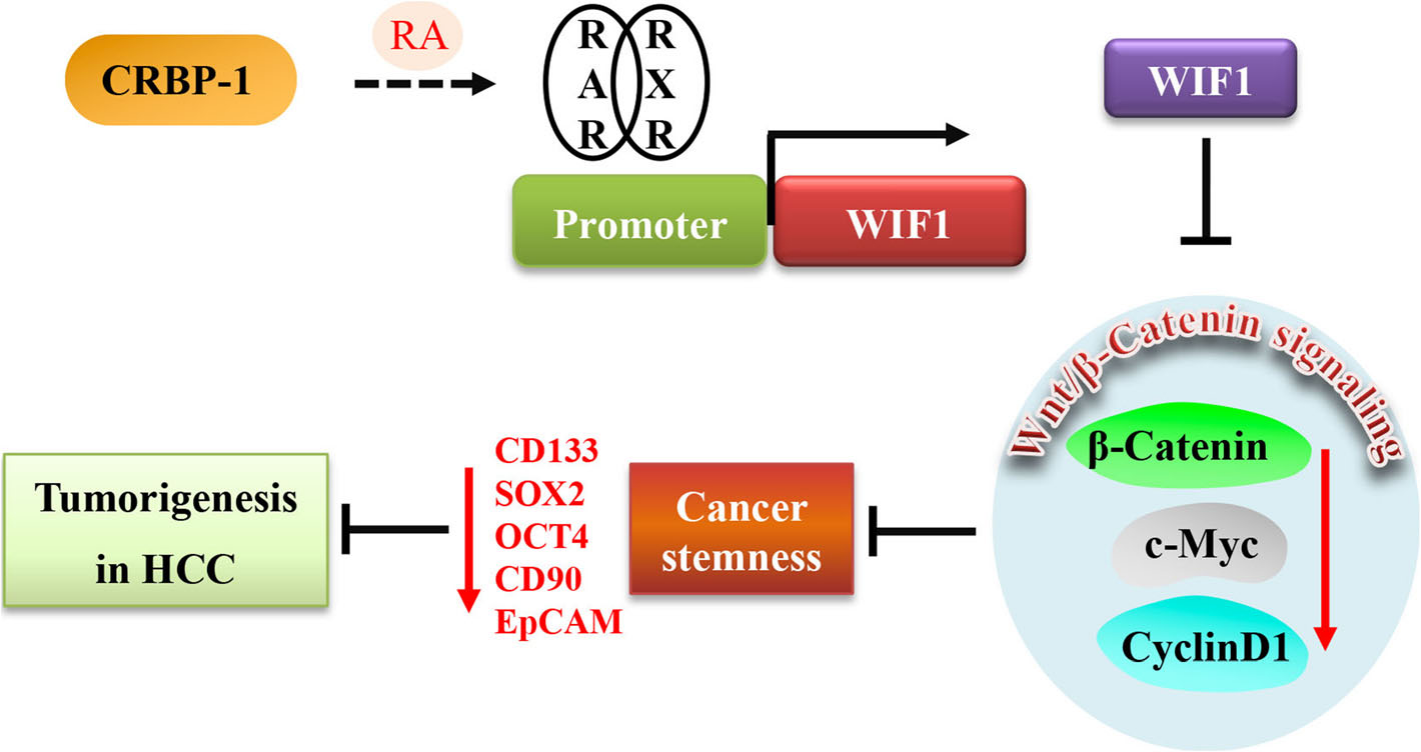
**Schematic illustration of CRBP-1 inhibiting cancer stemness via regulating wnt/β-catenin signaling pathway in HCC.** CRBP-1 induces the intracellular levels of RA, which activates RAR/RXR and transcriptionally promotes the expression of WIF1 via activating RAREs in its promoter region, thus downregulates the expression of β-catenin and its correlated genes, consequently suppress the cancer stemness properties leading to the inhibition of HCC tumorigenesis

In summary, our present study identified that CRBP-1 was downregulated in HCC tissues, and positively correlated with overall survival and recurrence free survival of HCC patients. Overexpression of CRBP-1 inhibited the cell growth and tumorigenicity both in vitro and in vivo. Furthermore, we found that CRBP-1 induced the intracellular levels of RA, which further activated transcriptional factor RARs/RXRs and transcriptionally promoted the expression of WIF1, thus enhancing the degradation of β-catenin, consequently suppressed the expression of cancer stemness related genes leading to the inhibition of HCC stemness properties (Fig. 6). Therefore, our findings provided a novel independent prognostic biomarker and therapeutic target for the diagnosis and treatment of HCC.

## Supplementary Information

**Additional file 1.**

## Data Availability

The datasets analyzed in the current study are available from the corresponding author on request.

## Abbreviations

CRBP-1: cellular retinol binding protein-1
HCC: hepatocellular carcinoma
CSCs: cancer stem cells
RA: retinoic acid
RARs: retinoic acid receptors
RXRs: retinoic x receptors
RARE: retinoic acid response element
TMAs: tissue microarrays
HRP: horseradish peroxidase
DAB: diaminobenzidine
TCGA: the cancer genome atlas
FPKM: fragments per kilobase of transcript per million mapped reads
GAPDH: glyceraldehyde-3-phosphate dehydrogenase
ChIP: chromatin immunoprecipitation
OR: odds ratio
CI: confidence intervals
OS: overall survival
SD: standard deviation
GSEA: gene set enrichment analysis
KEGG: kyoto encyclopedia of genes and genomes
AFP: alpha-fetoprotein
ALT: alanine transaminase
AST: aspartate transaminase
PLT: platelet
ALB: albumin
RFS: recurrence free survival
WIF1: Wnt inhibitory factor 1
RARβ: retinoic acid receptor β
CYP26A1: cytochrome P450 26A1
HOXa1: homeobox A1
APML: acute promyelocytic leukemia
RAS: retinoic acid syndrome

## References

[1] Batlle E, Clevers H (2017). Cancer stem cells revisited. Nat Med.

[2] Fiori ME, Villanova L, De Maria R (2017). Cancer stem cells: at the forefront of personalized medicine and immunotherapy. Curr Opin Pharmacol.

[3] Kim WT, Ryu CJ (2017). Cancer stem cell surface markers on normal stem cells. BMB Rep.

[4] Matsui WH (2016). Cancer stem cell signaling pathways. Medicine (Baltimore).

[5] Bray F, Ferlay J, Soerjomataram I, Siegel RL, Torre LA, Jemal A (2018). Global cancer statistics 2018: GLOBOCAN estimates of incidence and mortality worldwide for 36 cancers in 185 countries. CA Cancer J Clin.

[6] Yagci T, Cetin M, Ercin PB: Cancer Stem Cells in Hepatocellular Carcinoma. J Gastrointest Cancer 2017, 48(3), 48, 3, 245, DOI: 10.1007/s12029-017-9960-7. 10.1007/s12029-017-9960-7 28643126

[7] Suetsugu A, Nagaki M, Aoki H, Motohashi T, Kunisada T, Moriwaki H (2006). Characterization of CD133+ hepatocellular carcinoma cells as cancer stem/progenitor cells. Biochem Biophys Res Commun.

[8] Flores-Tellez TN, Villa-Trevino S, Pina-Vazquez C (2017). Road to stemness in hepatocellular carcinoma. World J Gastroenterol.

[9] Wang W, Smits R, Hao H, He C (2019). Wnt/beta-catenin signaling in liver cancers. Cancers (Basel).

[10] Mark M, Ghyselinck NB, Chambon P (2006). Function of retinoid nuclear receptors: lessons from genetic and pharmacological dissections of the retinoic acid signaling pathway during mouse embryogenesis. Annu Rev Pharmacol Toxicol.

[11] Altucci L, Gronemeyer H (2001). The promise of retinoids to fight against cancer. Nat Rev Cancer.

[12] Bushue N, Wan YJ (2010). Retinoid pathway and cancer therapeutics. Adv Drug Deliv Rev.

[13] Osei-Sarfo K, Gudas LJ (2014). Retinoic acid suppresses the canonical Wnt signaling pathway in embryonic stem cells and activates the noncanonical Wnt signaling pathway. Stem Cells.

[14] Zhu X, Wang W, Zhang X, Bai J, Chen G, Li L, Li M (2015). All-trans retinoic acid-induced deficiency of the Wnt/beta-catenin pathway enhances hepatic carcinoma stem cell differentiation. PLoS One.

[15] Napoli JL (2017). Cellular retinoid binding-proteins, CRBP, CRABP, FABP5: effects on retinoid metabolism, function and related diseases. Pharmacol Ther.

[16] Kuppumbatti YS, Bleiweiss IJ, Mandeli JP, Waxman S, Mira YLR (2000). Cellular retinol-binding protein expression and breast cancer. J Natl Cancer Inst.

[17] Orlandi A, Ferlosio A, Ciucci A, Francesconi A, Lifschitz-Mercer B, Gabbiani G, Spagnoli LG, Czernobilsky B (2006). Cellular retinol binding protein-1 expression in endometrial hyperplasia and carcinoma: diagnostic and possible therapeutic implications. Mod Pathol.

[18] Schmitt-Graff A, Ertelt V, Allgaier HP, Koelble K, Olschewski M, Nitschke R, Bochaton-Piallat ML, Gabbiani G, Blum HE (2003). Cellular retinol-binding protein-1 in hepatocellular carcinoma correlates with beta-catenin, Ki-67 index, and patient survival. Hepatology.

[19] Cvetkovic D, Williams SJ, Hamilton TC (2003). Loss of cellular retinol-binding protein 1 gene expression in microdissected human ovarian cancer. Clinical cancer research : an official journal of the American Association for Cancer Research.

[20] Jeronimo C, Henrique R, Oliveira J, Lobo F, Pais I, Teixeira MR, Lopes C (2004). Aberrant cellular retinol binding protein 1 (CRBP1) gene expression and promoter methylation in prostate cancer. J Clin Pathol.

[21] Campos B, Centner FS, Bermejo JL, Ali R, Dorsch K, Wan F, Felsberg J, Ahmadi R, Grabe N, Reifenberger G, Unterberg A, Burhenne J, Herold-Mende C (2011). Aberrant expression of retinoic acid signaling molecules influences patient survival in astrocytic gliomas. Am J Pathol.

[22] Doldo E, Costanza G, Ferlosio A, Pompeo E, Agostinelli S, Bellezza G, Mazzaglia D, Giunta A, Sidoni A, Orlandi A (2015). High expression of cellular retinol binding protein-1 in lung adenocarcinoma is associated with poor prognosis. Genes Cancer.

[23] Peralta R, Baudis M, Vazquez G, Juarez S, Ortiz R, Decanini H, Hernandez D, Gallegos F, Valdivia A, Pina P (2010). Increased expression of cellular retinol-binding protein 1 in laryngeal squamous cell carcinoma. J Cancer Res Clin Oncol.

[24] Chen Y, Tian T, Mao MJ, Deng WY, Li H (2018). CRBP-1 over-expression is associated with poor prognosis in tongue squamous cell carcinoma. BMC Cancer.

[25] Gao L, Wang Q, Ren W, Zheng J, Li S, Dou Z, Kong X, Liang X, Zhi K (2020). The RBP1-CKAP4 axis activates oncogenic autophagy and promotes cancer progression in oral squamous cell carcinoma. Cell Death Dis.

[26] Hoegberg P, Schmidt CK, Fletcher N, Nilsson CB, Trossvik C, Gerlienke Schuur A, Brouwer A, Nau H, Ghyselinck NB, Chambon P, Håkansson H (2005). Retinoid status and responsiveness to 2,3,7,8-tetrachlorodibenzo-p-dioxin (TCDD) in mice lacking retinoid binding protein or retinoid receptor forms. Chem Biol Interact.

[27] Pierzchalski K, Yu J, Norman V, Kane MA (2013). CrbpI regulates mammary retinoic acid homeostasis and the mammary microenvironment. FASEB J.

[28] Gao X, Shan W, Liu X, Zhang J, Zheng J, Yao H (2018). JNK1/2 and EKR1/2 provides vital clues about tumor recurrence and survival in hepatocellular carcinoma patients. Future Oncol.

[29] Livak KJ, Schmittgen TD (2001). Analysis of relative gene expression data using real-time quantitative PCR and the 2(−Delta Delta C(T)) method. Methods.

[30] Hou P, Li L, Chen F, Chen Y, Liu H, Li J, Bai J, Zheng J (2018). PTBP3-mediated regulation of ZEB1 mRNA stability promotes epithelial-mesenchymal transition in breast Cancer. Cancer Res.

[31] Trapnell C, Roberts A, Goff L, Pertea G, Kim D, Kelley DR, Pimentel H, Salzberg SL, Rinn JL, Pachter L (2012). Differential gene and transcript expression analysis of RNA-seq experiments with TopHat and cufflinks. Nat Protoc.

[32] Ayob AZ, Ramasamy TS (2018). Cancer stem cells as key drivers of tumour progression. J Biomed Sci.

[33] Zhang J, Zhou B, Liu Y, Chen K, Bao P, Wang Y, Wang J, Zhou Z, Sun X, Li Y (2014). Wnt inhibitory factor-1 functions as a tumor suppressor through modulating Wnt/beta-catenin signaling in neuroblastoma. Cancer Lett.

[34] Malinauskas T, Jones EY (2014). Extracellular modulators of Wnt signalling. Curr Opin Struct Biol.

[35] Li VS, Ng SS, Boersema PJ, Low TY, Karthaus WR, Gerlach JP, Mohammed S, Heck AJ, Maurice MM, Mahmoudi T (2012). Wnt signaling through inhibition of beta-catenin degradation in an intact Axin1 complex. Cell.

[36] Tang XH, Gudas LJ (2011). Retinoids, retinoic acid receptors, and cancer. Annu Rev Pathol.

[37] Srivastava J, Robertson CL, Rajasekaran D, Gredler R, Siddiq A, Emdad L, Mukhopadhyay ND, Ghosh S, Hylemon PB, Gil G, Shah K, Bhere D, Subler MA, Windle JJ, Fisher PB, Sarkar D (2014). AEG-1 regulates retinoid X receptor and inhibits retinoid signaling. Cancer Res.

[38] Yagci T, Cetin M, Ercin PB: Cancer Stem Cells in Hepatocellular Carcinoma. *J Gastrointest Cancer* 2017, Cancer Stem Cells in Hepatocellular Carcinoma. 10.1007/s12029-017-9960-7 28643126

[39] Tallman MS, Andersen JW, Schiffer CA, Appelbaum FR, Feusner JH, Woods WG, Ogden A, Weinstein H, Shepherd L, Willman C, Bloomfield CD, Rowe JM, Wiernik PH (2002). All-trans retinoic acid in acute promyelocytic leukemia: long-term outcome and prognostic factor analysis from the north American intergroup protocol. Blood.

[40] Patatanian E, Thompson DF (2008). Retinoic acid syndrome: a review. J Clin Pharm Ther.

[41] Wang T, Ma X, Krausz KW, Idle JR, Gonzalez FJ (2008). Role of pregnane X receptor in control of all-trans retinoic acid (ATRA) metabolism and its potential contribution to ATRA resistance. J Pharmacol Exp Ther.

[42] Moise AR, Noy N, Palczewski K, Blaner WS (2007). Delivery of retinoid-based therapies to target tissues. Biochemistry.

[43] Castelli G, Pelosi E, Testa U (2017). Liver Cancer: molecular characterization, clonal evolution and Cancer stem cells. Cancers (Basel).

[44] Chiba T, Iwama A, Yokosuka O (2016). Cancer stem cells in hepatocellular carcinoma: therapeutic implications based on stem cell biology. Hepatol Res.

[45] Clevers H, Nusse R (2012). Wnt/beta-catenin signaling and disease. Cell.

[46] Wang W, Smits R, Hao H, He C: Wnt/beta-Catenin Signaling in Liver Cancers. Cancers (Basel) 2019, 11(7), Wnt/β-Catenin Signaling in Liver Cancers, 11, 7, DOI: 10.3390/cancers11070926. 10.3390/cancers11070926 PMC6679127 31269694

[47] Russell JO, Monga SP (2018). Wnt/beta-catenin signaling in liver development, homeostasis, and pathobiology. Annu Rev Pathol.

[48] He TC, Sparks AB, Rago C, Hermeking H, Zawel L, da Costa LT, Morin PJ, Vogelstein B, Kinzler KW (1998). Identification of c-MYC as a target of the APC pathway. Science.

[49] Tetsu O, McCormick F (1999). Beta-catenin regulates expression of cyclin D1 in colon carcinoma cells. Nature.

[50] Xu L, Song C, Ni M, Meng F, Xie H, Li G (2012). Cellular retinol-binding protein 1 (CRBP-1) regulates osteogenenesis and adipogenesis of mesenchymal stem cells through inhibiting RXRalpha-induced beta-catenin degradation. Int J Biochem Cell Biol.

[51] Mazieres J, He B, You L, Xu Z, Lee AY, Mikami I, Reguart N, Rosell R, McCormick F, Jablons DM (2004). Wnt inhibitory factor-1 is silenced by promoter hypermethylation in human lung cancer. Cancer Res.

[52] Ramachandran I, Thavathiru E, Ramalingam S, Natarajan G, Mills WK, Benbrook DM, Zuna R, Lightfoot S, Reis A, Anant S, Queimado L (2012). Wnt inhibitory factor 1 induces apoptosis and inhibits cervical cancer growth, invasion and angiogenesis in vivo. Oncogene.

[53] Rubin EM, Guo Y, Tu K, Xie J, Zi X, Hoang BH (2010). Wnt inhibitory factor 1 decreases tumorigenesis and metastasis in osteosarcoma. Mol Cancer Ther.

[54] Kim SA, Kwak J, Nam HY, Chun SM, Lee BW, Lee HJ, Khang SK, Kim SW (2013). Promoter methylation of WNT inhibitory factor-1 and expression pattern of WNT/beta-catenin pathway in human astrocytoma: pathologic and prognostic correlations. Mod Pathol.

[55] Ramachandran I, Ganapathy V, Gillies E, Fonseca I, Sureban SM, Houchen CW, Reis A, Queimado L (2014). Wnt inhibitory factor 1 suppresses cancer stemness and induces cellular senescence. Cell Death Dis.

