# Supplementary material for: Cellular retinol binding protein-1 inhibits cancer stemness via upregulating WIF1 to suppress Wnt/β-catenin pathway in hepatocellular carcinoma
Source: BMC Cancer. 2021 Nov 14;21:1224. doi: 10.1186/s12885-021-08967-2 (PMC8590789; doi:10.1186/s12885-021-08967-2)
Supplement: Supplementary file 1 — Additional file 1. [file 12885_2021_8967_MOESM1_ESM.docx]

**Title: Cellular retinol binding protein-1 inhibits cancer stemness via upregulating WIF1 to suppress Wnt/β-catenin pathway in hepatocellular carcinoma**

**Running title: CRBP-1 inhibits CSCs via regulating WIF1/Wnt/β-catenin pathway in HCC**

Xiangye Liu^1, #, *^, Wenhua Shan^2, *^, Tingting Li^1, *^, Xiaoge Gao^2^, Fanyun Kong^1^, Hongjuan You^1^, Delong Kong^1^, Shuxi Qiao^2^, Renxian Tang^1, #^

1. Jiangsu Key Laboratory of Immunity and Metabolism, Department of Pathogenic Biology and Immunology, Xuzhou Medical University, Xuzhou, Jiangsu Province, 221004, P. R. China

2. Cancer Institute, Xuzhou Medical University, Xuzhou, Jiangsu Province, 221002, P. R. China

# Correspondence to:

Dr. Xiangye Liu, Jiangsu Key Laboratory of Immunity and Metabolism, Department of Pathogenic Biology and Immunology, Xuzhou Medical University, Xuzhou, Jiangsu Province, 221004, P. R. China, liuxy83@xzhmu.edu.cn;

Prof. Renxian Tang, Jiangsu Key Laboratory of Immunity and Metabolism, Department of Pathogenic Biology and Immunology, Xuzhou Medical University, Xuzhou, Jiangsu Province, 221004, P. R. China, tangrenxian-t@163.com

* Xiangye Liu, Wenhua Shan, and Tingting Li contributed equally to this work.

**Supplementary tables**

**Table S1. The nucleotide sequences used in the present study**

| **Gene name** | **Forward (5′ to 3′)** | **Reverse (5′ to 3′)** | **Applications** |
| --- | --- | --- | --- |
| **CRBP-1** | AATGTGGCCTTGCGCAAAAT | CAGCTCATCACCCTCGATCC | qPCR |
| **CD133** | CACTACCAAGGACAAGGCGTTC | CAACGCCTCTTTGGTCTCCTTG | qPCR |
| **OCT4** | CCTGAAGCAGAAGAGGATCACC | AAAGCGGCAGATGGTCGTTTGG | qPCR |
| **SOX2** | GCTACAGCATGATGCAGGACCA | TCTGCGAGCTGGTCATGGAGTT | qPCR |
| **WIF1** | TCAGAAAAGCGCAACAGAGA | TGATGCCTTTATCCAGGGAG | qPCR |
| **CTNNB1** | TTCGAAATCTTGCCCTTTGTCCCG | AATTCGGTTGTGAACATCCCGAGC | qPCR |
| **HOXa1** | TCCTGGAATACCCCATACTTAGCA | GCCGCCGCAACTGTTG | qPCR |
| **CYP26A1** | CGAGCACTCGTGGGAGAG | CCAAAGAGGAGTTCGGTTGA | qPCR |
| **RARB2** | GATTGACCCAAACCGAATGGCAGCA | ATTTGTCCTGGCAGACGAAGCA | qPCR |
| **GAPDH** | AATCCCATCACCATCTTCC | CATCACGCCACAGTTTCC | qPCR |
| **c-Myc** | CCTGGTGCTCCATGAGGAGAC | CAGACTCTGACCTTTTGCCAGG | qPCR |
| **CCND1** | TCTACACCGACAACTCCATCCG | TCTGGCATTTTGGAGAGGAAGTG | qPCR |
| **RARE1** | TGTGAGGATTAAGTAAGCAC | GCTATACAACCAACCAATAG | PCR |
| **RARE2** | AGCGCCGGAGGAGGAACA | AGACAACGCTGGGCTTGAC | PCR |
| **pCDH-CRBP-1** | ATATCTAGAATGGATCCTCCCGCAGGCT | GCGGAATTCTCACTGCACCTTCTTGAATACTTG | Plasmid construction |
| **pGL4.20-WIF1** | ATAGCTAGCTCTGCTGGGCCAGGTGGATAG | TATAAGCTTTGCTGCTCAGGACCTCCTCGCTG | Plasmid construction |

**Table S2. Differential expressed genes (fold change ≥ 1.5, *p* < 0.05) in PLC/PRF/5 cells in response to CRBP-1 overexpression.** In total of 78 genes were upregulated and 158 were downregulated. The genes in red color were cancer stemness related one, which have been reported.

| **Gene ID** | **Gene Name** | **Locus** | **Con_FPKM** | **CRBP-1_FPKM** | **Fold change** | **Up / down** | ***p* value** | **Description** |
| --- | --- | --- | --- | --- | --- | --- | --- | --- |
| ENSG00000114115 | CRBP-1 | 3:139389814-139678017 | 0.466935 | 127.956 | 8.09822 | up | 0 | retinol binding protein 1 [Source:HGNC Symbol;Acc:HGNC:9919] |
| ENSG00000263934 | SNORD3A | 17:19188015-19188714 | 0.165099 | 26.6993 | 7.33733 | up | 3.44E-12 | - |
| ENSG00000274944 | RP5-864K19.6 | 1:38838197-38941799 | 0.0183125 | 0.913544 | 5.64057 | up | 0.00549017 | - |
| ENSG00000167779 | IGFBP6 | 12:53097435-53102345 | 0.0272713 | 0.72239 | 4.72732 | up | 0.00172747 | insulin like growth factor binding protein 6 [Source:HGNC Symbol;Acc:HGNC:5475] |
| ENSG00000278952 | RP11-399J13.2 | 11:65118309-65119111 | 0.143782 | 1.78934 | 3.63747 | up | 0.00077399 | - |
| ENSG00000156076 | WIF1 | 12:65017467-65121566 | 0.0703676 | 0.793853 | 3.49589 | up | 0.000239853 | WNT inhibitory factor 1 [Source:HGNC Symbol;Acc:HGNC:18081] |
| ENSG00000274012 | RN7SL2 | 14:49861175-49864379 | 18.7145 | 205.985 | 3.46031 | up | 0 | - |
| ENSG00000263740 | RN7SL4P | 3:15667235-15859771 | 0.934153 | 9.85631 | 3.39932 | up | 1.03E-05 | - |
| ENSG00000223956 | RP4-710M16.2 | 1:56414962-56415966 | 0.217904 | 2.14987 | 3.30248 | up | 0.00245478 | - |
| ENSG00000103426 | CORO7-PAM16 | 16:4314760-4425705 | 0.0738399 | 0.648053 | 3.13364 | up | 0.000328508 | CORO7-PAM16 readthrough [Source:HGNC Symbol;Acc:HGNC:44424] |
| ENSG00000134240 | HMGCS2 | 1:119747995-119768905 | 0.241849 | 1.9212 | 2.98983 | up | 2.58E-08 | 3-hydroxy-3-methylglutaryl-CoA synthase 2 [Source:HGNC Symbol;Acc:HGNC:5008] |
| ENSG00000172733 | PURG | 8:30995801-31033715 | 0.0616055 | 0.476378 | 2.95098 | up | 0.000546915 | purine rich element binding protein G [Source:HGNC Symbol;Acc:HGNC:17930] |
| ENSG00000269900 | RMRP | 9:35657750-35658018 | 2.4197 | 17.4417 | 2.84964 | up | 0.000151875 | - |
| ENSG00000202198 | 7SK | 6:52995619-52995950 | 6.47324 | 43.6695 | 2.75407 | up | 9.40E-10 | - |
| ENSG00000272369 | RP11-446N19.1 | 12:46383678-46876159 | 0.509266 | 3.39984 | 2.73898 | up | 0.00508217 | - |
| ENSG00000278771 | Metazoa_SRP | 14:49853615-49853914 | 1.45848 | 9.53623 | 2.70896 | up | 5.88E-06 | - |
| ENSG00000122711 | SPINK4 | 9:33218364-33264761 | 0.597083 | 3.85083 | 2.68917 | up | 0.00225849 | serine peptidase inhibitor, Kazal type 4 [Source:HGNC Symbol;Acc:HGNC:16646] |
| ENSG00000279148 | RP11-135F9.4 | 12:100026247-100027540 | 0.135772 | 0.83326 | 2.61758 | up | 0.00449017 | - |
| ENSG00000151790 | TDO2 | 4:155829728-155920406 | 0.277198 | 1.70076 | 2.61719 | up | 4.51E-05 | tryptophan 2,3-dioxygenase [Source:HGNC Symbol;Acc:HGNC:11708] |
| ENSG00000163009 | C2orf48 | 2:10141381-10211725 | 0.0842713 | 0.493183 | 2.54901 | up | 0.00165485 | - |
| ENSG00000109181 | UGT2B10 | 4:68815992-68831196 | 0.43619 | 2.53764 | 2.54046 | up | 2.49E-06 | UDP glucuronosyltransferase family 2 member B10 [Source:HGNC Symbol;Acc:HGNC:12544] |
| ENSG00000258472 | RP11-192H23.4 | 17:28455751-28645454 | 0.12057 | 0.693878 | 2.52481 | up | 0.000499847 | - |
| ENSG00000250186 | RP11-1079K10.4 | 17:49370739-49476988 | 0.332941 | 1.88911 | 2.50437 | up | 0.00397772 | - |
| ENSG00000148795 | CYP17A1 | 10:102830530-102837533 | 0.124478 | 0.615015 | 2.30473 | up | 0.00181826 | cytochrome P450 family 17 subfamily A member 1 [Source:HGNC Symbol;Acc:HGNC:2593] |
| ENSG00000203811 | HIST2H3C | 1:149832658-149841193 | 0.172489 | 0.851746 | 2.30392 | up | 0.000374573 | histone cluster 2 H3 family member c [Source:HGNC Symbol;Acc:HGNC:20503] |
| ENSG00000259112 | NDUFC2-KCTD14 | 11:78015714-78080219 | 3.87295 | 18.8433 | 2.28255 | up | 2.62E-07 | NDUFC2-KCTD14 readthrough [Source:HGNC Symbol;Acc:HGNC:42956] |
| ENSG00000184709 | LRRC26 | 9:137138389-137172409 | 0.271937 | 1.27671 | 2.23109 | up | 0.00012757 | leucine rich repeat containing 26 [Source:HGNC Symbol;Acc:HGNC:31409] |
| ENSG00000224287 | MSL3P1 | 2:233865436-233868444 | 0.291275 | 1.36603 | 2.22954 | up | 0.000711656 | male-specific lethal 3 homolog (Drosophila) pseudogene 1 [Source:HGNC Symbol;Acc:HGNC:17837] |
| ENSG00000198610 | AKR1C4 | 10:5195461-5218949 | 0.153402 | 0.71597 | 2.22259 | up | 0.00552643 | aldo-keto reductase family 1 member C4 [Source:HGNC Symbol;Acc:HGNC:387] |
| ENSG00000213759 | UGT2B11 | 4:69181659-69216766 | 1.4551 | 6.62018 | 2.18576 | up | 6.78E-08 | UDP glucuronosyltransferase family 2 member B11 [Source:HGNC Symbol;Acc:HGNC:12545] |
| ENSG00000281383 | CH507-513H4.5 | 21:8254591-8255514 | 671.31 | 3012.11 | 2.16572 | up | 1.96E-12 | - |
| ENSG00000249863 | RP11-177C12.1 | 4:37868291-37869978 | 0.285094 | 1.23761 | 2.11805 | up | 0.00451021 | - |
| ENSG00000144045 | DQX1 | 2:74518130-74526336 | 0.281421 | 1.2135 | 2.10837 | up | 4.75E-06 | DEAQ-box RNA dependent ATPase 1 [Source:HGNC Symbol;Acc:HGNC:20410] |
| ENSG00000189057 | FAM111B | 11:59107184-59127410 | 2.02587 | 8.67894 | 2.09898 | up | 0 | family with sequence similarity 111 member B [Source:HGNC Symbol;Acc:HGNC:24200] |
| ENSG00000174992 | ZG16 | 16:29778239-29782973 | 0.136941 | 0.57817 | 2.07794 | up | 0.000369023 | zymogen granule protein 16 [Source:HGNC Symbol;Acc:HGNC:30961] |
| ENSG00000112299 | VNN1 | 6:132681589-132714049 | 0.173296 | 0.731563 | 2.07775 | up | 0.000369489 | vanin 1 [Source:HGNC Symbol;Acc:HGNC:12705] |
| ENSG00000107159 | CA9 | 9:35658289-35681159 | 1.99345 | 8.36217 | 2.06861 | up | 1.14E-10 | carbonic anhydrase 9 [Source:HGNC Symbol;Acc:HGNC:1383] |
| ENSG00000180592 | SKIDA1 | 10:21513477-21743630 | 0.208471 | 0.873115 | 2.06632 | up | 2.51E-06 | SKI/DACH domain containing 1 [Source:HGNC Symbol;Acc:HGNC:32697] |
| ENSG00000116194 | ANGPTL1 | 1:178724305-178921841 | 0.355807 | 1.48575 | 2.06203 | up | 2.58E-05 | angiopoietin like 1 [Source:HGNC Symbol;Acc:HGNC:489] |
| ENSG00000261040 | WFDC21P | 17:60079308-60091885 | 1.09114 | 4.55204 | 2.06067 | up | 0.000482493 | - |
| ENSG00000280385 | AP000648.5 | 11:90193613-90198120 | 0.286226 | 1.18923 | 2.0548 | up | 0.000148995 | - |
| ENSG00000000971 | CFH | 1:196651877-196747504 | 0.481852 | 1.99618 | 2.05058 | up | 1.25E-06 | complement factor H [Source:HGNC Symbol;Acc:HGNC:4883] |
| ENSG00000279377 | AC003973.3 | 19:21932957-22010949 | 0.191377 | 0.730983 | 1.93342 | up | 0.002131 | - |
| ENSG00000198788 | MUC2 | 11:1074874-1110511 | 0.306392 | 1.15061 | 1.90895 | up | 3.62E-05 | - |
| ENSG00000203852 | HIST2H3A | 1:149852618-149861210 | 0.195133 | 0.729419 | 1.90229 | up | 0.00223367 | histone cluster 2 H3 family member a [Source:HGNC Symbol;Acc:HGNC:20505] |
| ENSG00000163586 | FABP1 | 2:88122981-88128116 | 31.457 | 117.313 | 1.8989 | up | 2.12351E-10 | fatty acid binding protein 1 [Source:HGNC Symbol;Acc:HGNC:3555] |
| ENSG00000272384 | RP11-44N11.2 | 8:122779970-122781071 | 0.494629 | 1.82208 | 1.88117 | up | 0.00175727 | - |
| ENSG00000204388 | HSPA1B | 6:31827734-31830255 | 9.95111 | 36.3906 | 1.87064 | up | 2.13E-11 | heat shock protein family A (Hsp70) member 1B [Source:HGNC Symbol;Acc:HGNC:5233] |
| ENSG00000219891 | ZSCAN12P1 | 6:28091153-28093664 | 0.237266 | 0.856479 | 1.85191 | up | 0.00393699 | zinc finger and SCAN domain containing 12 pseudogene 1 [Source:HGNC Symbol;Acc:HGNC:13850] |
| ENSG00000281181 | CH507-513H4.3 | 21:8437628-8438551 | 1516.58 | 5431.64 | 1.84056 | up | 5.03E-08 | - |
| ENSG00000181856 | SLC2A4 | 17:7281666-7294615 | 0.314907 | 1.12448 | 1.83626 | up | 9.05E-05 | solute carrier family 2 member 4 [Source:HGNC Symbol;Acc:HGNC:11009] |
| ENSG00000135346 | CGA | 6:87085497-87095406 | 3.76212 | 13.3418 | 1.82634 | up | 2.33E-06 | glycoprotein hormones, alpha polypeptide [Source:HGNC Symbol;Acc:HGNC:1885] |
| ENSG00000109971 | HSPA8 | 11:123057488-123063230 | 281.899 | 978.143 | 1.79486 | up | 0 | heat shock protein family A (Hsp70) member 8 [Source:HGNC Symbol;Acc:HGNC:5241] |
| ENSG00000225329 | LHFPL3-AS2 | 7:104328655-104926645 | 0.691814 | 2.37779 | 1.78116 | up | 0.000326281 | - |
| ENSG00000170516 | COX7B2 | 4:46734826-46909235 | 3.62868 | 12.3691 | 1.76923 | up | 8.39E-05 | cytochrome c oxidase subunit 7B2 [Source:HGNC Symbol;Acc:HGNC:24381] |
| ENSG00000148965 | SAA4 | 11:18231348-18248643 | 5.13699 | 17.4701 | 1.7659 | up | 7.22E-06 | serum amyloid A4, constitutive [Source:HGNC Symbol;Acc:HGNC:10516] |
| ENSG00000213213 | CCDC183 | 9:136791354-136841187 | 0.349261 | 1.15448 | 1.72486 | up | 0.00473888 | coiled-coil domain containing 183 [Source:HGNC Symbol;Acc:HGNC:28236] |
| ENSG00000283149 | RP11-134F2.8 | 3:186538440-186773476 | 2.11425 | 6.96657 | 1.72031 | up | 0.00593979 | - |
| ENSG00000165376 | CLDN2 | X:106802679-107000244 | 2.5855 | 8.49024 | 1.71536 | up | 1.64E-09 | claudin 2 [Source:HGNC Symbol;Acc:HGNC:2041] |
| ENSG00000271430 | RP3-368A4.5 | X:73944323-74293574 | 0.767827 | 2.51669 | 1.71268 | up | 7.83E-05 | - |
| ENSG00000236901 | MIR600HG | 9:123109493-123268576 | 0.215712 | 0.704018 | 1.7065 | up | 0.00015126 | - |
| ENSG00000128242 | GAL3ST1 | 22:30554634-30574587 | 1.26443 | 4.09201 | 1.69432 | up | 4.89E-07 | galactose-3-O-sulfotransferase 1 [Source:HGNC Symbol;Acc:HGNC:24240] |
| ENSG00000204389 | HSPA1A | 6:31815463-31817946 | 15.1051 | 48.8673 | 1.69384 | up | 0 | heat shock protein family A (Hsp70) member 1A [Source:HGNC Symbol;Acc:HGNC:5232] |
| ENSG00000056291 | NPFFR2 | 4:72031803-72148067 | 0.677887 | 2.1915 | 1.6928 | up | 0.00018023 | neuropeptide FF receptor 2 [Source:HGNC Symbol;Acc:HGNC:4525] |
| ENSG00000073849 | ST6GAL1 | 3:186930484-187078553 | 0.36558 | 1.18092 | 1.69165 | up | 0.000157238 | ST6 beta-galactoside alpha-2,6-sialyltransferase 1 [Source:HGNC Symbol;Acc:HGNC:10860] |
| ENSG00000036473 | OTC | X:37349274-38688920 | 0.990441 | 3.13065 | 1.66032 | up | 0.000411156 | ornithine carbamoyltransferase [Source:HGNC Symbol;Acc:HGNC:8512] |
| ENSG00000180739 | S1PR5 | 19:10512741-10517931 | 0.918149 | 2.89351 | 1.65602 | up | 1.05E-05 | sphingosine-1-phosphate receptor 5 [Source:HGNC Symbol;Acc:HGNC:14299] |
| ENSG00000241635 | UGT1A1 | 2:233617644-233773310 | 4.84298 | 14.9496 | 1.62614 | up | 3.72E-07 | UDP glucuronosyltransferase family 1 member A1 [Source:HGNC Symbol;Acc:HGNC:12530] |
| ENSG00000275740 | RP11-449H3.3 | 5:146203549-146376963 | 0.296759 | 0.915279 | 1.62492 | up | 0.00375526 | - |
| ENSG00000173930 | SLCO4C1 | 5:102233985-102296549 | 0.159987 | 0.490925 | 1.61754 | up | 0.00296401 | solute carrier organic anion transporter family member 4C1 [Source:HGNC Symbol;Acc:HGNC:23612] |
| ENSG00000174844 | DNAH12 | 3:57293698-57544344 | 1.20852 | 3.5889 | 1.57029 | up | 4.86E-06 | dynein axonemal heavy chain 12 [Source:HGNC Symbol;Acc:HGNC:2943] |
| ENSG00000121858 | TNFSF10 | 3:172505507-172523507 | 4.98495 | 14.7921 | 1.56918 | up | 8.76E-07 | tumor necrosis factor superfamily member 10 [Source:HGNC Symbol;Acc:HGNC:11925] |
| ENSG00000105538 | RASIP1 | 19:48720586-48740721 | 0.213468 | 0.632349 | 1.5667 | up | 0.0056917 | Ras interacting protein 1 [Source:HGNC Symbol;Acc:HGNC:24716] |
| ENSG00000114268 | PFKFB4 | 3:48517683-48563773 | 1.02609 | 3.0209 | 1.55783 | up | 1.06E-05 | 6-phosphofructo-2-kinase/fructose-2,6-biphosphatase 4 [Source:HGNC Symbol;Acc:HGNC:8875] |
| ENSG00000215472 | RPL17-C18orf32 | 18:49477249-49492523 | 4.0891 | 11.9322 | 1.54501 | up | 0.000224695 | RPL17-C18orf32 readthrough [Source:HGNC Symbol;Acc:HGNC:44661] |
| ENSG00000187758 | ADH1A | 4:99088856-99301356 | 0.84366 | 2.43636 | 1.52999 | up | 0.00183854 | alcohol dehydrogenase 1A (class I), alpha polypeptide [Source:HGNC Symbol;Acc:HGNC:249] |
| ENSG00000130600 | H19 | 11:1995162-2001470 | 8.90077 | 25.4225 | 1.51411 | up | 1.33E-15 | - |
| ENSG00000005981 | ASB4 | 7:95478443-95540232 | 1.12005 | 3.19248 | 1.51111 | up | 7.60E-05 | ankyrin repeat and SOCS box containing 4 [Source:HGNC Symbol;Acc:HGNC:16009] |
| ENSG00000248866 | USP46-AS1 | 4:52659405-52661668 | 2.95238 | 1.04135 | -1.50342 | down | 0.0012739 | - |
| ENSG00000079156 | OSBPL6 | 2:178194480-178402891 | 5.31679 | 1.86535 | -1.51111 | down | 4.51E-12 | oxysterol binding protein like 6 [Source:HGNC Symbol;Acc:HGNC:16388] |
| ENSG00000088826 | SMOX | 20:4120979-4187747 | 28.8072 | 10.0057 | -1.5256 | down | 0 | spermine oxidase [Source:HGNC Symbol;Acc:HGNC:15862] |
| ENSG00000043039 | BARX2 | 11:129375939-129452279 | 1.80099 | 0.624934 | -1.52701 | down | 0.000566507 | BARX homeobox 2 [Source:HGNC Symbol;Acc:HGNC:956] |
| ENSG00000232593 | KANTR | X:53093709-53167014 | 1.79189 | 0.619406 | -1.53252 | down | 0.00300957 | - |
| ENSG00000163545 | NUAK2 | 1:205302058-205321791 | 17.8977 | 6.18484 | -1.53297 | down | 8.61E-08 | NUAK family kinase 2 [Source:HGNC Symbol;Acc:HGNC:29558] |
| ENSG00000186868 | MAPT | 17:45620327-46028334 | 0.822961 | 0.284163 | -1.53411 | down | 0.00162906 | microtubule associated protein tau [Source:HGNC Symbol;Acc:HGNC:6893] |
| ENSG00000258701 | LINC00638 | 14:104821200-104823718 | 1.96877 | 0.677565 | -1.53886 | down | 0.000256286 | - |
| ENSG00000272502 | RP11-713M15.2 | 8:120380760-120813359 | 13.9043 | 4.76965 | -1.54358 | down | 1.54E-05 | - |
| ENSG00000147576 | ADHFE1 | 8:66429027-66518524 | 2.58291 | 0.885626 | -1.54423 | down | 0.000342379 | alcohol dehydrogenase, iron containing 1 [Source:HGNC Symbol;Acc:HGNC:16354] |
| ENSG00000100290 | BIK | 22:43110747-43129712 | 2.24458 | 0.769523 | -1.54441 | down | 0.00289376 | BCL2 interacting killer [Source:HGNC Symbol;Acc:HGNC:1051] |
| ENSG00000127528 | KLF2 | 19:16324816-16327874 | 2.93087 | 1.00438 | -1.54502 | down | 8.79E-05 | Kruppel like factor 2 [Source:HGNC Symbol;Acc:HGNC:6347] |
| ENSG00000254876 | RP11-23J9.5 | 9:97156569-97378524 | 1.0594 | 0.362364 | -1.54773 | down | 0.00133012 | - |
| ENSG00000214575 | CPEB1 | 15:82536752-82709914 | 5.76474 | 1.97139 | -1.54804 | down | 4.60E-10 | cytoplasmic polyadenylation element binding protein 1 [Source:HGNC Symbol;Acc:HGNC:21744] |
| ENSG00000127585 | FBXL16 | 16:684621-705829 | 0.796349 | 0.272076 | -1.54939 | down | 1.35E-05 | F-box and leucine rich repeat protein 16 [Source:HGNC Symbol;Acc:HGNC:14150] |
| ENSG00000261641 | LA16c-390E6.5 | 16:1444933-1475580 | 2.52451 | 0.85588 | -1.56052 | down | 0.00438629 | - |
| ENSG00000128849 | CGNL1 | 15:57375966-57550727 | 0.651556 | 0.220699 | -1.56181 | down | 0.000179021 | cingulin like 1 [Source:HGNC Symbol;Acc:HGNC:25931] |
| ENSG00000006652 | IFRD1 | 7:112422967-112491062 | 81.8137 | 27.6477 | -1.56518 | down | 6.24E-11 | interferon related developmental regulator 1 [Source:HGNC Symbol;Acc:HGNC:5456] |
| ENSG00000074935 | TUBE1 | 6:112054071-112087529 | 17.6296 | 5.93464 | -1.57076 | down | 1.73E-07 | tubulin epsilon 1 [Source:HGNC Symbol;Acc:HGNC:20775] |
| ENSG00000180938 | ZNF572 | 8:124973297-124979389 | 0.66512 | 0.223634 | -1.57248 | down | 0.0043886 | zinc finger protein 572 [Source:HGNC Symbol;Acc:HGNC:26758] |
| ENSG00000267221 | CTD-2132N18.2 | 17:41966740-42098479 | 0.553226 | 0.18515 | -1.57917 | down | 0.00181573 | - |
| ENSG00000135069 | PSAT1 | 9:78297142-78330093 | 284.745 | 94.804 | -1.58665 | down | 1.37E-09 | phosphoserine aminotransferase 1 [Source:HGNC Symbol;Acc:HGNC:19129] |
| ENSG00000134463 | ECHDC3 | 10:11742365-11764070 | 1.91213 | 0.635026 | -1.59029 | down | 0.000422456 | enoyl-CoA hydratase domain containing 3 [Source:HGNC Symbol;Acc:HGNC:23489] |
| ENSG00000279809 | AC005538.3 | 2:233865436-233868444 | 1.9584 | 0.648942 | -1.59352 | down | 0.00323873 | - |
| ENSG00000106366 | SERPINE1 | 7:101127088-101139266 | 3.20199 | 1.06045 | -1.5943 | down | 1.21E-05 | serpin family E member 1 [Source:HGNC Symbol;Acc:HGNC:8583] |
| ENSG00000157693 | TMEM268 | 9:114611205-114646422 | 11.9233 | 3.93209 | -1.60041 | down | 1.74E-11 | transmembrane protein 268 [Source:HGNC Symbol;Acc:HGNC:24513] |
| ENSG00000179148 | ALOXE3 | 17:8095899-8119047 | 0.671124 | 0.220376 | -1.60662 | down | 0.00133154 | arachidonate lipoxygenase 3 [Source:HGNC Symbol;Acc:HGNC:13743] |
| ENSG00000065911 | MTHFD2 | 2:74198561-74421662 | 134.115 | 43.6216 | -1.62035 | down | 8.88E-16 | methylenetetrahydrofolate dehydrogenase (NADP+ dependent) 2, methenyltetrahydrofolate cyclohydrolase [Source:HGNC Symbol;Acc:HGNC:7434] |
| ENSG00000179598 | PLD6 | 17:17042544-17237188 | 2.52581 | 0.820618 | -1.62196 | down | 4.92E-05 | phospholipase D family member 6 [Source:HGNC Symbol;Acc:HGNC:30447] |
| ENSG00000101384 | JAG1 | 20:10637683-10994924 | 37.5896 | 12.1012 | -1.63519 | down | 5.23E-10 | jagged 1 [Source:HGNC Symbol;Acc:HGNC:6188] |
| ENSG00000163633 | C4orf36 | 4:86876204-87141054 | 1.53108 | 0.492128 | -1.63744 | down | 0.000940426 | chromosome 4 open reading frame 36 [Source:HGNC Symbol;Acc:HGNC:28386] |
| ENSG00000175592 | FOSL1 | 11:65892048-65900573 | 10.7904 | 3.4594 | -1.64116 | down | 2.53E-12 | FOS like 1, AP-1 transcription factor subunit [Source:HGNC Symbol;Acc:HGNC:13718] |
| ENSG00000234741 | GAS5 | 1:173863247-173903549 | 181.735 | 58.1624 | -1.64368 | down | 0 | - |
| ENSG00000139289 | PHLDA1 | 12:76025446-76033932 | 57.1688 | 18.2649 | -1.64615 | down | 2.44E-12 | pleckstrin homology like domain family A member 1 [Source:HGNC Symbol;Acc:HGNC:8933] |
| ENSG00000140044 | JDP2 | 14:75423682-75474111 | 10.1262 | 3.22064 | -1.65267 | down | 1.40E-09 | Jun dimerization protein 2 [Source:HGNC Symbol;Acc:HGNC:17546] |
| ENSG00000177989 | ODF3B | 22:50523567-50532580 | 1.82147 | 0.578488 | -1.65475 | down | 0.000171883 | outer dense fiber of sperm tails 3B [Source:HGNC Symbol;Acc:HGNC:34388] |
| ENSG00000178093 | TSSK6 | 19:19512417-19515685 | 4.05603 | 1.28425 | -1.65914 | down | 4.05E-09 | testis specific serine kinase 6 [Source:HGNC Symbol;Acc:HGNC:30410] |
| ENSG00000204387 | C6orf48 | 6:31834607-31839766 | 127.599 | 40.3077 | -1.66249 | down | 0 | chromosome 6 open reading frame 48 [Source:HGNC Symbol;Acc:HGNC:19078] |
| ENSG00000223802 | CERS1 | 19:18868544-18896727 | 4.30539 | 1.35872 | -1.6639 | down | 3.97E-07 | ceramide synthase 1 [Source:HGNC Symbol;Acc:HGNC:14253] |
| ENSG00000174564 | IL20RB | 3:136862207-137011085 | 0.697067 | 0.219717 | -1.66565 | down | 0.00278592 | interleukin 20 receptor subunit beta [Source:HGNC Symbol;Acc:HGNC:6004] |
| ENSG00000166192 | SENP8 | 15:71818395-72155459 | 0.883127 | 0.277287 | -1.67124 | down | 0.00065214 | SUMO/sentrin peptidase family member, NEDD8 specific [Source:HGNC Symbol;Acc:HGNC:22992] |
| ENSG00000275807 | RP11-1348G14.8 | 16:28822430-28837237 | 0.639767 | 0.198903 | -1.68548 | down | 0.0053543 | - |
| ENSG00000157601 | MX1 | 21:41420303-41459214 | 1.4208 | 0.441043 | -1.68772 | down | 6.26E-05 | MX dynamin like GTPase 1 [Source:HGNC Symbol;Acc:HGNC:7532] |
| ENSG00000257027 | RP11-705C15.3 | 12:9658566-9662085 | 0.793051 | 0.245981 | -1.68887 | down | 0.00474852 | - |
| ENSG00000177410 | ZFAS1 | 20:49219294-49484297 | 77.0971 | 23.9035 | -1.68946 | down | 0 | - |
| ENSG00000258927 | RP11-1070N10.5 | 14:95620913-95643285 | 0.967956 | 0.299846 | -1.69072 | down | 0.0039888 | - |
| ENSG00000248049 | UBA6-AS1 | 4:67701279-68129880 | 7.55252 | 2.3186 | -1.7037 | down | 2.36E-08 | - |
| ENSG00000103257 | SLC7A5 | 16:87830022-87869488 | 341.079 | 104.685 | -1.70405 | down | 0 | solute carrier family 7 member 5 [Source:HGNC Symbol;Acc:HGNC:11063] |
| ENSG00000128965 | CHAC1 | 15:40952961-40956519 | 79.5882 | 24.353 | -1.70845 | down | 1.25E-11 | ChaC glutathione specific gamma-glutamylcyclotransferase 1 [Source:HGNC Symbol;Acc:HGNC:28680] |
| ENSG00000142279 | WTIP | 19:34481637-34512304 | 6.10443 | 1.86045 | -1.7142 | down | 5.55E-07 | Wilms tumor 1 interacting protein [Source:HGNC Symbol;Acc:HGNC:20964] |
| ENSG00000186074 | CD300LF | 17:74670577-74769353 | 0.717867 | 0.218364 | -1.71698 | down | 0.00307644 | CD300 molecule like family member f [Source:HGNC Symbol;Acc:HGNC:29883] |
| ENSG00000072163 | LIMS2 | 2:127638380-127681786 | 1.74414 | 0.527837 | -1.72435 | down | 2.12E-05 | LIM zinc finger domain containing 2 [Source:HGNC Symbol;Acc:HGNC:16084] |
| ENSG00000171044 | XKR6 | 8:10896044-11201366 | 2.11331 | 0.636762 | -1.73068 | down | 0.000122193 | XK related 6 [Source:HGNC Symbol;Acc:HGNC:27806] |
| ENSG00000267530 | AC006273.5 | 19:782754-785080 | 13.2935 | 3.98873 | -1.73671 | down | 4.21E-08 | - |
| ENSG00000171951 | SCG2 | 2:223596939-223602503 | 1.80001 | 0.533886 | -1.75341 | down | 8.00E-05 | secretogranin II [Source:HGNC Symbol;Acc:HGNC:10575] |
| ENSG00000133134 | BEX2 | X:103309345-103311046 | 11.6899 | 3.46606 | -1.7539 | down | 1.19E-07 | brain expressed X-linked 2 [Source:HGNC Symbol;Acc:HGNC:30933] |
| ENSG00000105327 | BBC3 | 19:47220821-47232766 | 4.45436 | 1.31868 | -1.75613 | down | 7.74E-11 | BCL2 binding component 3 [Source:HGNC Symbol;Acc:HGNC:17868] |
| ENSG00000211958 | IGHV3-38 | 14:106410492-106411021 | 4.63545 | 1.36327 | -1.76563 | down | 0.00298416 | immunoglobulin heavy variable 3-38 (non-functional) [Source:HGNC Symbol;Acc:HGNC:5601] |
| ENSG00000254806 | SYS1-DBNDD2 | 20:45361936-45410610 | 1.85416 | 0.544856 | -1.76682 | down | 0.00207282 | SYS1-DBNDD2 readthrough (NMD candidate) [Source:HGNC Symbol;Acc:HGNC:33535] |
| ENSG00000100889 | PCK2 | 14:24080106-24132849 | 121.695 | 35.6645 | -1.77071 | down | 0 | phosphoenolpyruvate carboxykinase 2, mitochondrial [Source:HGNC Symbol;Acc:HGNC:8725] |
| ENSG00000151012 | SLC7A11 | 4:138027421-138242349 | 17.2803 | 5.05174 | -1.77428 | down | 4.50E-10 | solute carrier family 7 member 11 [Source:HGNC Symbol;Acc:HGNC:11059] |
| ENSG00000100814 | CCNB1IP1 | 14:20311367-20333312 | 59.2099 | 17.1419 | -1.78831 | down | 0 | cyclin B1 interacting protein 1 [Source:HGNC Symbol;Acc:HGNC:19437] |
| ENSG00000265972 | TXNIP | 1:145992434-145996600 | 13.6676 | 3.95547 | -1.78883 | down | 1.93E-08 | thioredoxin interacting protein [Source:HGNC Symbol;Acc:HGNC:16952] |
| ENSG00000129474 | AJUBA | 14:22929608-22982642 | 119.993 | 34.4776 | -1.79922 | down | 0 | ajuba LIM protein [Source:HGNC Symbol;Acc:HGNC:20250] |
| ENSG00000151025 | GPR158 | 10:25113057-25602226 | 3.07272 | 0.881881 | -1.80086 | down | 3.23E-07 | G protein-coupled receptor 158 [Source:HGNC Symbol;Acc:HGNC:23689] |
| ENSG00000257918 | RP11-482D24.3 | 12:106357657-106889316 | 2.62239 | 0.737831 | -1.82952 | down | 0.00168084 | - |
| ENSG00000259203 | RP11-209K10.2 | 15:52801613-52804942 | 5.702 | 1.60134 | -1.83219 | down | 0.000237263 | - |
| ENSG00000177606 | JUN | 1:58780787-58784327 | 24.1825 | 6.75797 | -1.8393 | down | 5.21E-11 | Jun proto-oncogene, AP-1 transcription factor subunit [Source:HGNC Symbol;Acc:HGNC:6204] |
| ENSG00000247844 | CCAT1 | 8:127207865-127219088 | 10.1024 | 2.8211 | -1.84036 | down | 1.07E-07 | - |
| ENSG00000105963 | ADAP1 | 7:897900-975599 | 1.91065 | 0.531963 | -1.84467 | down | 4.45E-06 | ArfGAP with dual PH domains 1 [Source:HGNC Symbol;Acc:HGNC:16486] |
| ENSG00000070669 | ASNS | 7:97852117-97872542 | 336.608 | 93.1188 | -1.85393 | down | 0 | asparagine synthetase (glutamine-hydrolyzing) [Source:HGNC Symbol;Acc:HGNC:753] |
| ENSG00000141391 | PRELID3A | 18:12407895-12432238 | 7.13648 | 1.96217 | -1.86276 | down | 1.90E-10 | PRELI domain containing 3A [Source:HGNC Symbol;Acc:HGNC:24639] |
| ENSG00000161270 | NPHS1 | 19:35825963-35879791 | 1.01863 | 0.279446 | -1.86599 | down | 2.76E-05 | NPHS1, nephrin [Source:HGNC Symbol;Acc:HGNC:7908] |
| ENSG00000139514 | SLC7A1 | 13:29509409-29595688 | 46.1384 | 12.6242 | -1.86978 | down | 4.25E-12 | solute carrier family 7 member 1 [Source:HGNC Symbol;Acc:HGNC:11057] |
| ENSG00000248498 | ASNSP1 | 8:46579212-46614523 | 0.948392 | 0.259423 | -1.87018 | down | 0.00342564 | asparagine synthetase pseudogene 1 [Source:HGNC Symbol;Acc:HGNC:754] |
| ENSG00000270194 | RP11-259K5.2 | 3:37241788-37366751 | 1.1981 | 0.327574 | -1.87085 | down | 0.000140017 | - |
| ENSG00000239467 | AC007405.6 | 2:170771112-170778148 | 4.6508 | 1.27134 | -1.87113 | down | 0.000291942 | - |
| ENSG00000246985 | SOCS2-AS1 | 12:93542462-93583487 | 2.16396 | 0.589568 | -1.87594 | down | 0.000283127 | - |
| ENSG00000229656 | RP11-462L8.1 | 10:32900318-33082102 | 9.84376 | 2.66588 | -1.8846 | down | 1.31E-06 | - |
| ENSG00000203865 | ATP1A1-AS1 | 1:116372667-116418622 | 2.34012 | 0.626248 | -1.90178 | down | 0.000621244 | - |
| ENSG00000110031 | LPXN | 11:58526870-58578220 | 2.20451 | 0.589612 | -1.90262 | down | 5.69E-06 | leupaxin [Source:HGNC Symbol;Acc:HGNC:14061] |
| ENSG00000155495 | MAGEC1 | X:141903893-141909388 | 0.942663 | 0.251351 | -1.90704 | down | 0.000577735 | MAGE family member C1 [Source:HGNC Symbol;Acc:HGNC:6812] |
| ENSG00000213988 | ZNF90 | 19:20077993-20321305 | 0.978453 | 0.260818 | -1.90746 | down | 0.00168088 | zinc finger protein 90 [Source:HGNC Symbol;Acc:HGNC:13165] |
| ENSG00000147852 | VLDLR | 9:2422701-2660053 | 1.309 | 0.341126 | -1.94009 | down | 6.72E-09 | very low density lipoprotein receptor [Source:HGNC Symbol;Acc:HGNC:12698] |
| ENSG00000189060 | H1F0 | 22:37805092-37807436 | 77.0204 | 20.0315 | -1.94297 | down | 8.85E-13 | H1 histone family member 0 [Source:HGNC Symbol;Acc:HGNC:4714] |
| ENSG00000154319 | FAM167A | 8:11339636-11474715 | 1.50843 | 0.388184 | -1.95824 | down | 1.30E-06 | family with sequence similarity 167 member A [Source:HGNC Symbol;Acc:HGNC:15549] |
| ENSG00000269893 | SNHG8 | 4:118278708-118279823 | 106.333 | 27.2734 | -1.96303 | down | 2.22E-16 | - |
| ENSG00000099284 | H2AFY2 | 10:70052795-70132934 | 0.990519 | 0.253825 | -1.96435 | down | 0.000213145 | H2A histone family member Y2 [Source:HGNC Symbol;Acc:HGNC:14453] |
| ENSG00000121413 | ZSCAN18 | 19:58059238-58118427 | 0.586808 | 0.149393 | -1.97377 | down | 0.00400149 | zinc finger and SCAN domain containing 18 [Source:HGNC Symbol;Acc:HGNC:21037] |
| ENSG00000176788 | BASP1 | 5:17065597-17276843 | 0.607181 | 0.154373 | -1.97571 | down | 0.000434518 | brain abundant membrane attached signal protein 1 [Source:HGNC Symbol;Acc:HGNC:957] |
| ENSG00000178607 | ERN1 | 17:64039141-64130819 | 17.9426 | 4.50622 | -1.9934 | down | 0 | endoplasmic reticulum to nucleus signaling 1 [Source:HGNC Symbol;Acc:HGNC:3449] |
| ENSG00000122787 | AKR1D1 | 7:138002323-138117986 | 0.668298 | 0.167785 | -1.99388 | down | 0.00138712 | aldo-keto reductase family 1 member D1 [Source:HGNC Symbol;Acc:HGNC:388] |
| ENSG00000163132 | MSX1 | 4:4859665-4863936 | 0.58855 | 0.147377 | -1.99765 | down | 0.000679515 | msh homeobox 1 [Source:HGNC Symbol;Acc:HGNC:7391] |
| ENSG00000230409 | TCEA1P2 | 3:37241788-37366751 | 1.32744 | 0.32726 | -2.02013 | down | 0.00393979 | transcription elongation factor A1 pseudogene 2 [Source:HGNC Symbol;Acc:HGNC:29891] |
| ENSG00000142871 | CYR61 | 1:85580760-85583962 | 45.1159 | 11.1215 | -2.02028 | down | 9.31E-13 | cysteine rich angiogenic inducer 61 [Source:HGNC Symbol;Acc:HGNC:2654] |
| ENSG00000116717 | GADD45A | 1:67685060-67688338 | 9.22131 | 2.27251 | -2.02068 | down | 9.46E-10 | growth arrest and DNA damage inducible alpha [Source:HGNC Symbol;Acc:HGNC:4095] |
| ENSG00000229970 | AC007128.1 | 7:8113183-8344516 | 0.791185 | 0.194783 | -2.02215 | down | 0.00181386 | - |
| ENSG00000232956 | SNHG15 | 7:44983022-44986961 | 37.234 | 9.11222 | -2.03075 | down | 2.22E-16 | - |
| ENSG00000152503 | TRIM36 | 5:115124761-115180546 | 1.54143 | 0.376164 | -2.03483 | down | 2.04E-07 | tripartite motif containing 36 [Source:HGNC Symbol;Acc:HGNC:16280] |
| ENSG00000128165 | ADM2 | 22:50481555-50486440 | 7.47671 | 1.81466 | -2.0427 | down | 1.84E-11 | adrenomedullin 2 [Source:HGNC Symbol;Acc:HGNC:28898] |
| ENSG00000168003 | SLC3A2 | 11:62856101-62888875 | 511.863 | 122.833 | -2.05906 | down | 0 | solute carrier family 3 member 2 [Source:HGNC Symbol;Acc:HGNC:11026] |
| ENSG00000239969 | RP11-163E9.2 | 7:102363871-102426676 | 2.38253 | 0.567086 | -2.07085 | down | 0.000878559 | - |
| ENSG00000067840 | PDZD4 | X:153802165-153830565 | 1.97759 | 0.467201 | -2.08163 | down | 1.78E-15 | PDZ domain containing 4 [Source:HGNC Symbol;Acc:HGNC:21167] |
| ENSG00000120833 | SOCS2 | 12:93542462-93583487 | 11.6597 | 2.73932 | -2.08964 | down | 0 | suppressor of cytokine signaling 2 [Source:HGNC Symbol;Acc:HGNC:19382] |
| ENSG00000049249 | TNFRSF9 | 1:7915893-7943165 | 2.55478 | 0.599591 | -2.09115 | down | 2.31E-08 | TNF receptor superfamily member 9 [Source:HGNC Symbol;Acc:HGNC:11924] |
| ENSG00000107249 | GLIS3 | 9:3824126-4348392 | 9.31847 | 2.18486 | -2.09255 | down | 0 | GLIS family zinc finger 3 [Source:HGNC Symbol;Acc:HGNC:28510] |
| ENSG00000124143 | ARHGAP40 | 20:38601933-38651035 | 1.10249 | 0.258486 | -2.09261 | down | 6.85E-05 | Rho GTPase activating protein 40 [Source:HGNC Symbol;Acc:HGNC:16226] |
| ENSG00000136010 | ALDH1L2 | 12:104986309-105084577 | 0.840046 | 0.19161 | -2.1323 | down | 6.76E-08 | aldehyde dehydrogenase 1 family member L2 [Source:HGNC Symbol;Acc:HGNC:26777] |
| ENSG00000259040 | BLOC1S5-TXNDC5 | 6:7881516-8102578 | 4.71173 | 1.04812 | -2.16846 | down | 1.16E-07 | BLOC1S5-TXNDC5 readthrough (NMD candidate) [Source:HGNC Symbol;Acc:HGNC:42001] |
| ENSG00000130766 | SESN2 | 1:28259526-28282491 | 39.8084 | 8.7801 | -2.18077 | down | 3.33E-15 | sestrin 2 [Source:HGNC Symbol;Acc:HGNC:20746] |
| ENSG00000175197 | DDIT3 | 12:57460134-57520517 | 45.49 | 9.98429 | -2.18782 | down | 0 | DNA damage inducible transcript 3 [Source:HGNC Symbol;Acc:HGNC:2726] |
| ENSG00000113739 | STC2 | 5:173314712-173329503 | 38.4187 | 8.30317 | -2.21008 | down | 0 | stanniocalcin 2 [Source:HGNC Symbol;Acc:HGNC:11374] |
| ENSG00000131711 | MAP1B | 5:72107233-72209570 | 1.02667 | 0.218184 | -2.23435 | down | 5.37E-09 | microtubule associated protein 1B [Source:HGNC Symbol;Acc:HGNC:6836] |
| ENSG00000228782 | CTD-2026D20.3 | 17:47450567-47492492 | 2.94478 | 0.614664 | -2.26029 | down | 0.000109018 | - |
| ENSG00000167807 | CTD-2369P2.10 | 19:10304802-10339823 | 0.796417 | 0.164685 | -2.27381 | down | 0.000178002 | - |
| ENSG00000244459 | RP11-1398P2.1 | 4:1574061-1580253 | 1.50353 | 0.308759 | -2.2838 | down | 0.00265538 | - |
| ENSG00000136327 | NKX2-8 | 14:36320752-37173811 | 1.37334 | 0.277397 | -2.30767 | down | 5.27E-05 | NK2 homeobox 8 [Source:HGNC Symbol;Acc:HGNC:16364] |
| ENSG00000184860 | SDR42E1 | 16:81987947-82011488 | 0.740403 | 0.148868 | -2.31428 | down | 0.00440558 | short chain dehydrogenase/reductase family 42E, member 1 [Source:HGNC Symbol;Acc:HGNC:29834] |
| ENSG00000271888 | RP11-560J1.2 | 6:15243922-15245000 | 0.632479 | 0.125714 | -2.33088 | down | 0.00558091 | - |
| ENSG00000259319 | RP11-293M10.6 | 14:75423682-75474111 | 1.31511 | 0.250275 | -2.39359 | down | 1.63E-07 | - |
| ENSG00000250682 | LINC00491 | 5:102581367-102671559 | 2.88138 | 0.547277 | -2.39642 | down | 3.70E-06 | - |
| ENSG00000169554 | ZEB2 | 2:144384080-144524583 | 0.737611 | 0.139664 | -2.4009 | down | 3.20E-07 | zinc finger E-box binding homeobox 2 [Source:HGNC Symbol;Acc:HGNC:14881] |
| ENSG00000128564 | VGF | 7:101162508-101165593 | 0.569842 | 0.105852 | -2.42851 | down | 2.53E-05 | VGF nerve growth factor inducible [Source:HGNC Symbol;Acc:HGNC:12684] |
| ENSG00000145911 | N4BP3 | 5:178113442-178126087 | 0.744583 | 0.136225 | -2.45044 | down | 3.54E-08 | NEDD4 binding protein 3 [Source:HGNC Symbol;Acc:HGNC:29852] |
| ENSG00000105499 | PLA2G4C | 19:48047842-48110817 | 2.95097 | 0.538212 | -2.45494 | down | 3.05E-06 | phospholipase A2 group IVC [Source:HGNC Symbol;Acc:HGNC:9037] |
| ENSG00000048052 | HDAC9 | 7:18086948-19002416 | 1.21353 | 0.220856 | -2.45804 | down | 7.97E-08 | histone deacetylase 9 [Source:HGNC Symbol;Acc:HGNC:14065] |
| ENSG00000225361 | PPP1R26-AS1 | 9:135462726-135488893 | 0.798394 | 0.13871 | -2.52503 | down | 5.73E-05 | - |
| ENSG00000087074 | PPP1R15A | 19:48872391-48876057 | 54.7578 | 9.48406 | -2.52949 | down | 0 | protein phosphatase 1 regulatory subunit 15A [Source:HGNC Symbol;Acc:HGNC:14375] |
| ENSG00000255717 | SNHG1 | 11:62851987-62855914 | 306.803 | 51.4991 | -2.57469 | down | 0 | - |
| ENSG00000187479 | C11orf96 | 11:43921058-44001157 | 0.569641 | 0.0955804 | -2.57527 | down | 0.000597456 | chromosome 11 open reading frame 96 [Source:HGNC Symbol;Acc:HGNC:38675] |
| ENSG00000256955 | RP11-417L19.2 | 12:131857419-131864538 | 1.4848 | 0.246872 | -2.58844 | down | 0.000559912 | - |
| ENSG00000249345 | RP11-575F12.1 | 12:126915202-127060401 | 2.07894 | 0.339148 | -2.61586 | down | 0.000108159 | - |
| ENSG00000230647 | AC022816.2 | 17:14374138-14421026 | 0.886401 | 0.14292 | -2.63276 | down | 0.00520965 | - |
| ENSG00000107282 | APBA1 | 9:69427529-69672306 | 1.21718 | 0.191438 | -2.6686 | down | 2.16E-06 | amyloid beta precursor protein binding family A member 1 [Source:HGNC Symbol;Acc:HGNC:578] |
| ENSG00000114948 | ADAM23 | 2:206443538-206621130 | 0.796526 | 0.122372 | -2.70244 | down | 5.06E-05 | ADAM metallopeptidase domain 23 [Source:HGNC Symbol;Acc:HGNC:202] |
| ENSG00000106948 | AKNA | 9:114334155-114394405 | 5.51249 | 0.846028 | -2.70393 | down | 0 | AT-hook transcription factor [Source:HGNC Symbol;Acc:HGNC:24108] |
| ENSG00000196517 | SLC6A9 | 1:43991358-44115913 | 2.02178 | 0.306952 | -2.71954 | down | 1.23E-09 | solute carrier family 6 member 9 [Source:HGNC Symbol;Acc:HGNC:11056] |
| ENSG00000135842 | FAM129A | 1:184790723-184974550 | 1.85149 | 0.280607 | -2.72207 | down | 1.35E-11 | family with sequence similarity 129 member A [Source:HGNC Symbol;Acc:HGNC:16784] |
| ENSG00000139269 | INHBE | 12:57452322-57459280 | 25.8154 | 3.85402 | -2.7438 | down | 0 | inhibin beta E subunit [Source:HGNC Symbol;Acc:HGNC:24029] |
| ENSG00000095752 | IL11 | 19:55364388-55370463 | 1.71802 | 0.253455 | -2.76095 | down | 1.60E-08 | interleukin 11 [Source:HGNC Symbol;Acc:HGNC:5966] |
| ENSG00000087266 | SH3BP2 | 4:2793022-2841098 | 2.80759 | 0.392204 | -2.83966 | down | 2.91E-14 | SH3 domain binding protein 2 [Source:HGNC Symbol;Acc:HGNC:10825] |
| ENSG00000115902 | SLC1A4 | 2:64988476-65023865 | 21.9492 | 3.02916 | -2.85718 | down | 0 | solute carrier family 1 member 4 [Source:HGNC Symbol;Acc:HGNC:10942] |
| ENSG00000135116 | HRK | 12:116856143-116881441 | 1.26519 | 0.172492 | -2.87475 | down | 4.57E-10 | harakiri, BCL2 interacting protein [Source:HGNC Symbol;Acc:HGNC:5185] |
| ENSG00000123329 | ARHGAP9 | 12:57460134-57520517 | 0.851001 | 0.112638 | -2.91747 | down | 0.000608897 | Rho GTPase activating protein 9 [Source:HGNC Symbol;Acc:HGNC:14130] |
| ENSG00000101255 | TRIB3 | 20:362834-397559 | 78.9714 | 10.2811 | -2.94134 | down | 0 | tribbles pseudokinase 3 [Source:HGNC Symbol;Acc:HGNC:16228] |
| ENSG00000281028 | RP11-717K11.2 | 4:25160640-25279092 | 1.77376 | 0.228666 | -2.9555 | down | 0.00327166 | - |
| ENSG00000092969 | TGFB2 | 1:218346234-218444619 | 1.09222 | 0.131392 | -3.05532 | down | 3.78E-06 | transforming growth factor beta 2 [Source:HGNC Symbol;Acc:HGNC:11768] |
| ENSG00000167874 | TMEM88 | 17:7855064-7856099 | 0.721265 | 0.0772313 | -3.22327 | down | 0.00376235 | transmembrane protein 88 [Source:HGNC Symbol;Acc:HGNC:32371] |
| ENSG00000171462 | DLK2 | 6:43427365-43456632 | 1.25346 | 0.130429 | -3.26459 | down | 1.72E-06 | delta like non-canonical Notch ligand 2 [Source:HGNC Symbol;Acc:HGNC:21113] |
| ENSG00000234928 | AP000344.3 | 22:23433563-23435071 | 1.50623 | 0.150746 | -3.32076 | down | 0.000165556 | - |
| ENSG00000167995 | BEST1 | 11:61949820-61967660 | 1.36035 | 0.121374 | -3.48645 | down | 4.70E-13 | bestrophin 1 [Source:HGNC Symbol;Acc:HGNC:12703] |
| ENSG00000162772 | ATF3 | 1:212565333-212620777 | 32.5418 | 2.76897 | -3.55487 | down | 0 | activating transcription factor 3 [Source:HGNC Symbol;Acc:HGNC:785] |
| ENSG00000111981 | ULBP1 | 6:149964006-149973710 | 3.45549 | 0.273499 | -3.65928 | down | 2.00E-15 | UL16 binding protein 1 [Source:HGNC Symbol;Acc:HGNC:14893] |
| ENSG00000185633 | NDUFA4L2 | 12:57229326-57240715 | 0.601362 | 0.0459895 | -3.70886 | down | 0.00433433 | NDUFA4, mitochondrial complex associated like 2 [Source:HGNC Symbol;Acc:HGNC:29836] |
| ENSG00000170689 | HOXB9 | 17:48607226-48634932 | 0.647235 | 0.0435305 | -3.89419 | down | 8.83E-07 | homeobox B9 [Source:HGNC Symbol;Acc:HGNC:5120] |
| ENSG00000134470 | IL15RA | 10:5890202-5978187 | 1.11448 | 0.0664039 | -4.06896 | down | 8.27E-07 | interleukin 15 receptor subunit alpha [Source:HGNC Symbol;Acc:HGNC:5978] |
| ENSG00000149571 | KIRREL3 | 11:126355639-127006058 | 0.584492 | 0.0158346 | -5.20603 | down | 0.000390327 | kin of IRRE like 3 (Drosophila) [Source:HGNC Symbol;Acc:HGNC:23204] |
| ENSG00000188573 | FBLL1 | 5:168529115-168530634 | 0.977057 | 0.0157021 | -5.95942 | down | 5.51E-05 | fibrillarin-like 1 [Source:HGNC Symbol;Acc:HGNC:35458] |

**Table S3. Signaling pathways enriched with a *p*-value < 0.05 in HCC samples corresponding CRBP-1 expression by GSEA.**

| **NAME** | **SIZE** | **ES** | **NES** | **NOM *p*-value** |
| --- | --- | --- | --- | --- |
| KEGG_BASAL_CELL_CARCINOMA | 55 | -0.60298 | -1.90203 | 0 |
| KEGG_WNT_SIGNALING_PATHWAY | 150 | -0.56426 | -1.89909 | 0 |
| KEGG_VEGF_SIGNALING_PATHWAY | 76 | -0.54772 | -1.84983 | 0.001969 |
| KEGG_VASCULAR_SMOOTH_MUSCLE_CONTRACTION | 115 | -0.54513 | -1.84957 | 0.008097 |
| KEGG_THYROID_CANCER | 29 | -0.63022 | -1.82814 | 0.005917 |
| KEGG_AXON_GUIDANCE | 129 | -0.56601 | -1.79378 | 0.00616 |
| KEGG_NOTCH_SIGNALING_PATHWAY | 47 | -0.61988 | -1.78679 | 0.001996 |
| KEGG_ARRHYTHMOGENIC_RIGHT_VENTRICULAR_CARDIOMYOPATHY_ARVC | 74 | -0.58377 | -1.78452 | 0.002092 |
| KEGG_GNRH_SIGNALING_PATHWAY | 101 | -0.54065 | -1.78446 | 0.002075 |
| KEGG_HEDGEHOG_SIGNALING_PATHWAY | 56 | -0.56705 | -1.77821 | 0 |
| KEGG_MELANOGENESIS | 101 | -0.51324 | -1.73464 | 0.006198 |
| KEGG_PURINE_METABOLISM | 158 | -0.4744 | -1.72682 | 0.001923 |
| KEGG_ETHER_LIPID_METABOLISM | 33 | -0.5436 | -1.72233 | 0.004057 |
| KEGG_CALCIUM_SIGNALING_PATHWAY | 177 | -0.47352 | -1.71621 | 0.008065 |
| KEGG_GLYCOSPHINGOLIPID_BIOSYNTHESIS_GANGLIO_SERIES | 15 | -0.65549 | -1.71354 | 0.010163 |
| KEGG_SMALL_CELL_LUNG_CANCER | 84 | -0.55017 | -1.71337 | 0.01 |
| KEGG_MAPK_SIGNALING_PATHWAY | 267 | -0.48115 | -1.71305 | 0.01006 |
| KEGG_HYPERTROPHIC_CARDIOMYOPATHY_HCM | 83 | -0.5446 | -1.70979 | 0.00404 |
| KEGG_FOCAL_ADHESION | 199 | -0.53031 | -1.70594 | 0.020121 |
| KEGG_DILATED_CARDIOMYOPATHY | 90 | -0.54985 | -1.69968 | 0.006135 |
| KEGG_GAP_JUNCTION | 90 | -0.52961 | -1.67361 | 0.017682 |
| KEGG_ADHERENS_JUNCTION | 73 | -0.55434 | -1.66943 | 0.020202 |
| KEGG_TGF_BETA_SIGNALING_PATHWAY | 85 | -0.53226 | -1.65599 | 0.01 |
| KEGG_PATHWAYS_IN_CANCER | 325 | -0.47745 | -1.65599 | 0.011976 |
| KEGG_CHRONIC_MYELOID_LEUKEMIA | 73 | -0.53503 | -1.63351 | 0.033074 |
| KEGG_VASOPRESSIN_REGULATED_WATER_REABSORPTION | 44 | -0.56007 | -1.63086 | 0.021956 |
| KEGG_RNA_POLYMERASE | 29 | -0.61049 | -1.63002 | 0.021484 |
| KEGG_ECM_RECEPTOR_INTERACTION | 84 | -0.5373 | -1.61969 | 0.030738 |
| KEGG_T_CELL_RECEPTOR_SIGNALING_PATHWAY | 108 | -0.55141 | -1.61204 | 0.025 |
| KEGG_LONG_TERM_DEPRESSION | 70 | -0.47965 | -1.60674 | 0.021739 |
| KEGG_SPLICEOSOME | 127 | -0.60499 | -1.60388 | 0.030181 |
| KEGG_GLYCEROPHOSPHOLIPID_METABOLISM | 77 | -0.44588 | -1.5981 | 0.013889 |
| KEGG_REGULATION_OF_AUTOPHAGY | 35 | -0.49908 | -1.59388 | 0.027613 |
| KEGG_FC_EPSILON_RI_SIGNALING_PATHWAY | 79 | -0.48257 | -1.59166 | 0.026157 |
| KEGG_RIBOFLAVIN_METABOLISM | 16 | -0.59366 | -1.59 | 0.027344 |
| KEGG_NEUROACTIVE_LIGAND_RECEPTOR_INTERACTION | 271 | -0.42114 | -1.58646 | 0.004 |
| KEGG_NITROGEN_METABOLISM | 23 | -0.53983 | -1.58252 | 0.021195 |
| KEGG_GLYCOSAMINOGLYCAN_BIOSYNTHESIS_CHONDROITIN_SULFATE | 22 | -0.59748 | -1.57493 | 0.028 |
| KEGG_GLYCOSAMINOGLYCAN_BIOSYNTHESIS_HEPARAN_SULFATE | 26 | -0.56366 | -1.57261 | 0.028056 |
| KEGG_GLYCOSAMINOGLYCAN_DEGRADATION | 21 | -0.57231 | -1.56242 | 0.026157 |
| KEGG_ENDOCYTOSIS | 181 | -0.46607 | -1.55745 | 0.040161 |
| KEGG_MTOR_SIGNALING_PATHWAY | 52 | -0.50171 | -1.55724 | 0.039683 |
| KEGG_BASAL_TRANSCRIPTION_FACTORS | 35 | -0.5601 | -1.55282 | 0.016032 |
| KEGG_GLIOMA | 65 | -0.47767 | -1.53635 | 0.034483 |
| KEGG_TIGHT_JUNCTION | 132 | -0.43986 | -1.52381 | 0.036364 |
